# Degeneracy Explains Diversity in Interneuronal Regulation of Pattern Separation in Heterogeneous Dentate Gyrus Networks

**DOI:** 10.1093/function/zqaf035

**Published:** 2025-08-01

**Authors:** Sarang Saini, Rishikesh Narayanan

**Affiliations:** Cellular Neurophysiology Laboratory, Molecular Biophysics Unit, Indian Institute of Science, Bangalore 560012, India; Cellular Neurophysiology Laboratory, Molecular Biophysics Unit, Indian Institute of Science, Bangalore 560012, India

**Keywords:** complex systems, hippocampus, interneurons, network-to-network variability, neural-circuit heterogeneities

## Abstract

Pattern separation, the ability of a network to distinguish similar inputs by transforming them into distinct outputs, was postulated by the Marr-Albus theory to be realized by divergent feedforward excitatory connectivity. Yet, there is evidence for strong but differential regulation of pattern separation by local circuit connectivity. How do we reconcile the conflicting views on local-circuit regulation of pattern separation in circuits receiving divergent feedforward connectivity? Here, we quantitatively examined a population of heterogeneous dentate gyrus (DG) spiking networks where identically divergent feedforward connectivity was enforced. We generated 20 000 random DG networks constructed with thousands of functionally validated, heterogeneous single-neuron models of 4 different DG neuronal subtypes. We recorded network outputs to morphed sets of input patterns and applied quantitative metrics that we developed to assess pattern separation performance of each network. Surprisingly, only 47 of these 20 000 networks (0.23%) manifested effective pattern separation showing that divergent feedforward connectivity alone does not guarantee pattern separation. Instead, our analyses unveiled strong contributions from the 3 interneuron subtypes toward granule cell sparsity and pattern separation, with pronounced network-to-network variability in such contributions. We traced this variability to differences in local synaptic weights across pattern-separating networks, highlighting synaptic degeneracy as a key mechanism that explains diversity in interneuronal regulation of pattern separation. Finally, we found heterogeneous DG networks to be more resilient to synaptic jitter compared to their homogeneous counterparts. Together, our findings reconcile conflicting evidence by revealing degeneracy in DG circuits, whereby similar pattern separation efficacy can arise through diverse interactions among granule cells and interneurons.

## Graphical Abstract

**Graphical Abstract:**
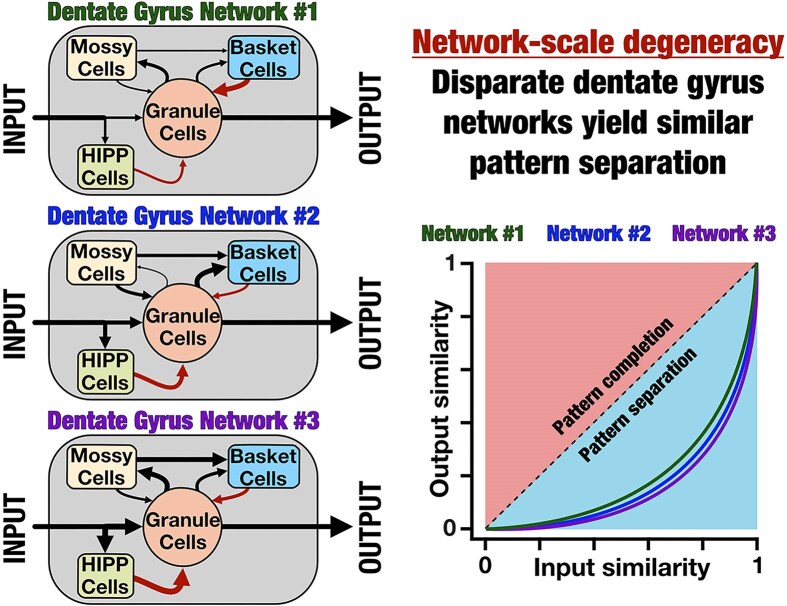

## Introduction

Pattern separation is the ability of a system to discriminate between similar inputs by transforming them into distinct outputs. In the context of the brain, inputs could originate from similar behavioral contexts or experiences and pattern separation is accomplished by neural circuits that transform these similar inputs into dissimilar neuronal outputs. The dentate gyrus (DG), the gateway to the hippocampus proper, is a well-studied pattern separation circuit that has also been implicated in memory formation, spatial navigation, and different learning paradigms. Historically, DG was postulated as a pattern-separating network based on divergent excitatory afferent connectivity from the entorhinal cortex.^1,2^ Since then, several studies have provided strong lines of evidence for a role of DG in pattern separation.^3-9^ Despite this, the mechanisms underlying pattern separation in the dentate gyrus have been highly debated,^10,11^ especially regarding the origins of sparse activity of granule cells,^12-16^ the roles of the several DG local-circuit components,^8,17-25^ implications for the widespread heterogeneities in the DG circuit,^26-28^ and the impact of adult neurogenesis and hyperplasticity on circuit formation.^9,29-35^ There are conflicting reports especially on the specific roles of the different interneurons in the intricately connected DG circuitry in pattern separation, sparsity, and other functions.^8,10-12,14,16-20,36-42^

What are the relative contributions of divergent feedforward connectivity and local circuit interactions to pattern separation? How do we reconcile the contrasting lines of evidence on interneuron regulation of pattern separation in circuits endowed with divergent feedforward connectivity? Is there a unifying framework that could synthesize these conflicting lines of evidence on the roles of different circuit components in executing pattern separation? In addressing these questions, we chose an exhaustive population-of-networks approach to study pattern separation in the heterogeneous DG circuit that was endowed with different neuronal subtypes. First, we generated tens of thousands of random spiking neuronal models to arrive at thousands of non-repeating single-neuron models of 4 different DG neuronal subtypes, each satisfying their respective functional characteristics. We connected these heterogeneous populations of neurons with subtype proportions and local connectivity that reflected the DG microcircuit.

In a second level of unbiased search, we generated 20 000 identical networks that differed from each other only in their synaptic weight values and surprisingly found only 47 of these networks to perform effective pattern separation. We found the synaptic weight values in these heterogeneous pattern-separating networks to span a large range with weak pairwise relationships between weight values across networks. Strikingly, our analyses unveiled pronounced network-to-network variability in the impact of deleting each interneuron subtype on pattern separation performance, sparsity, and firing rates. Finally, our analyses demonstrated that DG networks endowed with within-cell-type heterogeneities were more resilient to synaptic perturbations compared to their homogeneous counterparts.

Together, our population-of-networks approach showed that divergent connectivity of afferent inputs does not guarantee pattern separation in DG networks. Instead, we demonstrate strong yet variable roles for interneurons in implementing pattern separation. Importantly, our analyses unveil multi-scale degeneracy in DG circuits, whereby similar single-neuron function was achieved through disparate parametric combinations and similar pattern separation efficacy was implemented by disparate circuit interactions. Our demonstration that pattern separation emerged through interactions among several non-unique, non-random combinations of circuit elements strongly advocate the use of the complex adaptive systems framework to study DG function. Within this unifying framework, one of several combinations of different functionally specialized subsystems (the different neuronal and circuit elements) interact with each other to yield the *complex* DG network that adaptively implements effective pattern separation.

The presence of several functionally specialized subsystems and the ability of several non-random, non-unique combinations of these subsystems to interact toward executing precise pattern separation offers reconciliation to widespread variability reported on the contributions of different subsystems. Specifically, a complex system manifesting degeneracy is bound to exhibit widespread variability in the role of different subsystems, in a context- and system-dependent fashion. Thus, our analyses underscore the unifying capacity of the complex adaptive systems framework, strongly emphasizing the need to use a population-of-networks approach rather than deriving conclusions from a single network for all conclusions. The complex adaptive systems framework and the population-of-networks approach together offer an ideal route to account for the several DG circuit components and heterogeneities therein, toward implementing robust pattern separation through flexible mechanistic routes.

## Results

### Functionally Validated Heterogeneous Populations of Models for Each of the 4 Different Neuronal Subtypes

We generated 4 independent heterogeneous populations of granule cells (GC), basket cells (BC), mossy cells (MC), and HIPP cells (HC). Granule cells are the principal excitatory neurons in the dentate gyrus, implicated in spatial navigation, engram formation, and pattern separation.^3,5,14-16,43,44^ They receive inputs from the medial as well as lateral entorhinal cortices and project to CA3 via mossy fibers.^25,45^ Mossy cells are excitatory interneurons located in the hilus. They have been shown to exhibit multiple place field activity and are implicated in pattern separation.^15,36,39,46^ Basket cells are GABAergic interneurons that provide perisomatic inhibition to granule cells. They have been attributed roles in defining sparsity as well as in pattern separation.^11^Hilar perforant path-associated (HIPP) cells, another class of inhibitory interneurons, receive direct inputs from the entorhinal cortex through the perforant pathway. These interneurons target the distal dendrites of granule cells and have been shown to participate in modulating pattern separation and novelty detection.^20,47^

**Figure 1.**
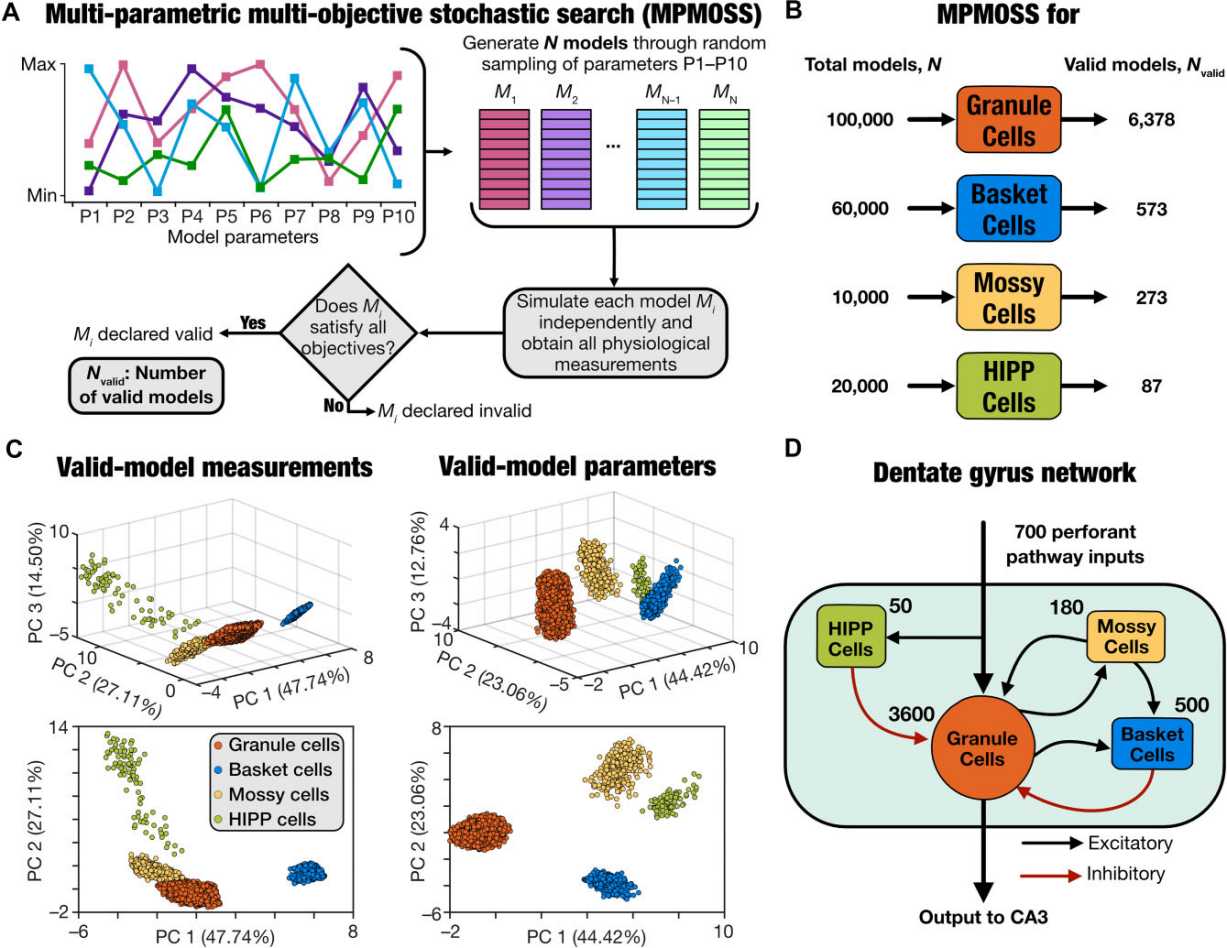
Dentate gyrus networks were constructed with cell-type-specific heterogeneous cell populations derived from independent unbiased stochastic searches. (A) Schematic of the MPMOSS algorithm used for generating heterogeneous populations of the 4 different cell types that were used to construct the heterogeneous dentate gyrus (DG) network. We used unbiased stochastic searches spanning a wide parametric space, independently performed for each cell type. For each cell type, the 9-dimensional parametric spaces of the aEIF models were randomly sampled (Supplementary Tables S1-S4) to yield $N$ individual models. Each randomly generated model was then validated against the physiological properties of the respective cell type (Supplementary Table S5). Models that satisfied all physiological properties for that cell type were declared to be valid, with ${{N}_{\textit{valid}}}$ representing the number of such valid models. (B) The total number of random models that were generated ($N$) and the number of valid models (${{N}_{\textit{valid}}}$) obtained for each of the 4 cell types. The number of valid models generated was constrained by the number of distinct models of each cell type required for generating the heterogeneous DG network. (C) Visualization of all valid models of the 4 different cell types (from panel B) on the same reduced 3D space for measurements (*left*) and parameters (*right*), through principal component analysis spanning all valid models. The clustering across and the heterogeneities within different cell types may be noted in both the measurement as well as parametric spaces. The numbers along each principal component (PC) axis indicates the percentage variance explained by that dimension. PC1 vs. PC2 is plotted below to demonstrate distinct clusters. (D) A graphical representation of the heterogeneous DG network architecture with 3600 unique granule cells and 3 unique sets of interneuron subtypes (500 basket, 180 mossy, and 50 HIPP cells) randomly picked from the respective heterogeneous population. The network receives inputs from the perforant pathway and sends its outputs to CA3 pyramidal cells.

We employed the well-established multi-parametric multi-objective stochastic search (MPMOSS) algorithm^26,34,48-56^ to generate valid heterogeneous populations of each cell type through independent and unbiased stochastic searches (Figure 1A). We used adaptive exponential integrate-and-fire (aEIF) spiking neuron models for all 4 neuronal subtypes. The search spanned the same set of 9 parameters, but with different ranges, for each of the different cell types (Supplementary Tables S1-S4). Randomly generated models were validated against subtype-specific signature electrophysiological measurements (Supplementary Table S5). We used 6 subtype-specific electrophysiological measurements for validation:^23,26,57-59^ membrane time constant (${{\tau }_m}$), sag ratio ($sag$), input resistance (${{R}_{in}}$), spike frequency adaptation ($\mathit{SFA}$), and action potential firing frequency for 50 ${\mathrm{pA}}$ (${{f}_{50}}$) or 150 ${\mathrm{pA}}$ (${{f}_{150}}$) pulse current injections. We randomly generated 100 000 granule cells, 60 000 basket cells, 10 000 mossy cells, and 20 000 HIPP cells from respective parametric distributions and validated them against their signature electrophysiological characteristics. Of these models, we found 6378 granule cells (∼6.3% of the total generated models), 573 basket cells (∼0.9%), 273 mossy cells (∼2.7%), and 87 HIPP cells (∼0.4%) to satisfy all electrophysiological constraints (Figure 1B).

We confirmed heterogeneities in the model parameters (Supplementary Figure S1) and physiological measurements (Supplementary Figure S2) of these models by noting the widespread nature of the ranges of each parameter and measurement, spanning their respective valid ranges in entirety. In addition, we plotted pairwise dependencies across model parameters (Supplementary Figure S1) and measurements (Supplementary Figure S2) and found most pairwise correlations to be weak. These observations are consistent with other computational and electrophysiological studies demonstrating degeneracy in different DG cell types,^26,34,54-57^ whereby disparate combinations of model parameters yield signature physiological outcomes. Importantly, since all 4 neuronal subtypes shared the same parametric and the measurements spaces, we performed principal component analysis on both spaces spanning all neuronal subtypes to visualize these models in reduced dimensions. We visualized all models as coefficients on the first 3 principal components, which explained ∼90% of the variance (Figure 1C). We found that the models belonging to the 4 neuronal subtypes formed distinct clusters while also manifesting heterogeneities within individual clusters (Figure 1C).

Together, these MPMOSS algorithms yielded heterogeneous populations of models that functionally matched each of the 4 neuronal subtypes. We used these single-neuron model populations to build a heterogeneous DG network (Figure 1D) that was made of non-repeating neurons with connectivity and synaptic properties (Supplementary Tables S6-S7) adopted from their biological counterparts.^23,37,47,58,59^ The network model thus was built with single-neuron models that matched the physiological heterogeneities of the different neuronal subtypes with local and afferent connectivity matching anatomical observations (Figure 1D).

### Progressively Morphed Patterns As Network Inputs

**Figure 2.**
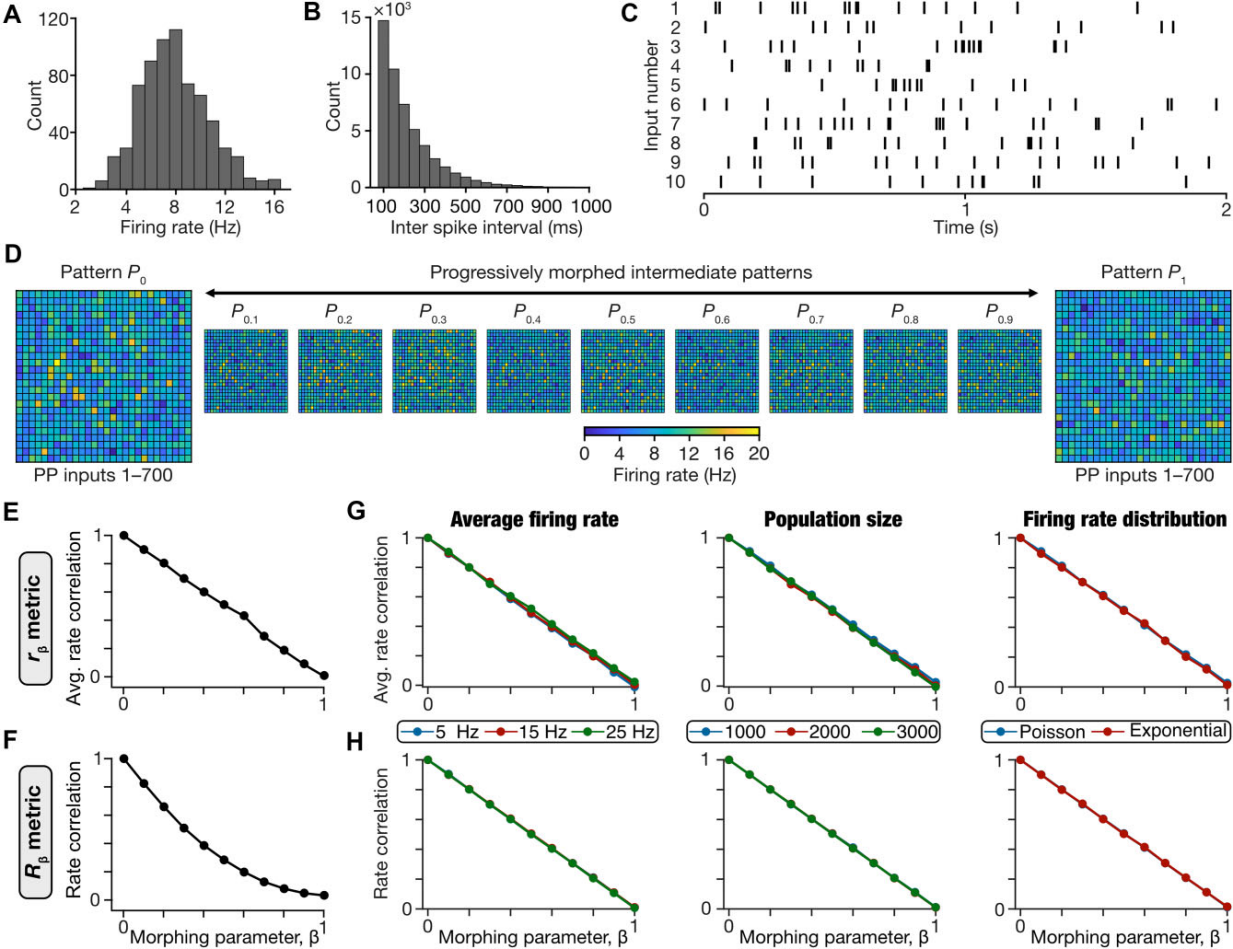
Input structure to the DG network and performance of different distance metrics. (A) Distribution of firing rates of 700 input neurons, derived from a Poisson distribution with a mean of 8 Hz. (B) Distribution of the inter-spike intervals generated from an exponential distribution with $\lambda $ of 125 ms. (C) Raster plot of randomly selected 10 out of 700 perforant pathway input neurons. (D) Two independent firing patterns, ${{P}_0}$ and ${{P}_1}$ were generated by randomly sampling the firing rate distribution. Each panel shows the heat map representing the average firing rate of 700 ($25\ \times \ 27$) neurons for both the patterns. ${{P}_0}$ was progressively morphed to ${{P}_1}$ to generate 9 intermediate patterns, ${{P}_\beta }$ with $0 < \ \beta \ < 1$. (E-F) Assessment of the correlation-based metrics for comparing the 11 different input patterns, with reference to the ${{P}_0}$ pattern. Shown are the computed similarity measures between ${{P}_\beta }$ (with $0 \le \ \beta \ \le 1$) and ${{P}_0}$ for the 2 pattern correlation metrics, average rate correlation $\ {{r}_\beta }$ (E) and rate correlation $\ {{R}_\beta }$ (F). (G-H) Performance of correlation-based metrics to quantify similarity of spike raster patterns generated for different average firing rate (*first column*), different population sizes (*second column*), and different firing rate distributions (*third column*). In each scenario, 10 sets of 2 distinct patterns (${{P}_0}$ and ${{P}_1}$) were generated to match the specifications (of firing rate, population size, and distribution type). For each of the 10 ${{P}_0}$–${{P}_1}$ pairs, 9 additional intermediate morphed patterns were created by morphing through ${{P}_0}$ to ${{P}_1}$. The metrics were computed for all patterns with reference to ${{P}_0}$ and the mean values across the 10 sets of patterns were plotted as functions of the morphing parameter ${\mathrm{\beta }}$. The metrics shown here are $\ {{r}_\beta }$ (G) and $\ {{R}_\beta }$ (H). The plots associated with panels (E-H) for the other metrics we tested are provided in Supplementary Figure S4.

The perforant pathway served as inputs to the DG network, with 700 afferent inputs projecting onto granule and HIPP cells within the network (Figure 1D). Each of these 700 PP inputs was modeled as an independent spike train, collectively forming one input pattern. The spike trains were randomly generated, with the spike frequency drawn from a Poisson distribution with $\lambda = 8\ {\mathrm{Hz}}$ (Figure 2A) and inter-spike intervals drawn from a corresponding exponential distribution (Figure 2B). A total of 700 spike trains that were constrained by these distributions (10 examples are shown in Figure 2C) were generated to yield one pattern (designated as ${{P}_0}$ in Figure 2D). A similar randomized generation process with a different seed yielded a second independent pattern (designated as ${{P}_1}$ in Figure 2D). These 2 patterns ${{P}_0}$ and ${{P}_1}$ served as extremes for generating 9 other morphed intermediate patterns that transitioned progressively from ${{P}_0}$ toward ${{P}_1}$ (Figure 2D). For a pattern ${{P}_\beta },\ \beta $ fraction of the total 700 spike trains were randomly selected from ${{P}_1}$ and the remaining ($1 - \beta )$ fraction from ${{P}_0}$. We varied β progressively from 0.1 to 0.9 in steps of 0.1, resulting in a total of 11 input patterns (${{P}_0},\ {{P}_{0.1}},\ {{P}_{0.2}},.\ .\ .,\ {{P}_{0.9}}\ $and ${{P}_1}$).

We assessed the similarity between ${{P}_0}$ and each of the 10 other input patterns using 6 different metrics: normalized Euclidean distance (Supplementary Figure S4*A*), Hamming distance (Supplementary Figure S4*B*), cosine similarity (Supplementary Figure S4*C*), mutual information (Supplementary Figure S4*D*), correlation between average firing rates, ${{r}_\beta }$ (Figure 2E), and the correlation between the instantaneous firing rates (${{R}_\beta }$) (Figure 2F; Supplementary Figure S3). As expected, the two distance-based similarity measurements increased as patterns diverged from each other (Supplementary Figure S4*A-B*), while cosine similarity, mutual information, and the correlation-based measures decreased with increasing dissimilarity (Supplementary Figure S4*C-D*; Figure 2E-F). The large distances between ${{P}_0}$ and ${{P}_1}$, coupled with the gradual changes in the measurement values as functions of the morphing parameter (Supplementary Figure S4*A-D*, Figure 2E-F) together confirmed that these patterns could be used to systematically assess pattern separation in our DG network using the morphed inputs approach.^3,60^

### Correlation-Based Metrics Were Invariant to Population Size, Average Firing Rate, and the Form of the Firing Rate Distribution of Input Patterns

If ${{S}_{in}}$ denoted similarity among the input patterns and ${{S}_{out}}$ represented the same similarity measure computed for the pattern-specific outputs of the network to these inputs, pattern separation was defined when ${{S}_{out}} < \ {{S}_{in}}$ across different levels of similarity among input patterns.^3,6,10,60-62^ We set 3 specific criteria in choosing the best of the several similarity measures across patterns made of spike trains (Figure 2G-H; Supplementary Figure S4*E-H*). First, consistent with the divergent anatomical connectivity in the DG network, our model consisted of 700 PP input spike trains and 3600 output spike trains from granule cells (Figure 1D). This difference in number of elements in the input vs. the output populations and the need to compare output vs. input similarity to quantitatively analyze pattern separation necessitated a metric that was *invariant to population size*. Second, as average firing rates of the input vs. output patterns could be very different, we required a metric that was *invariant to the average firing rates* of the population. Third, the firing rate distributions of the input and the output need not obey specific parametrized forms, especially given the sparse nature of GC firing.^13-16,63-65^ Therefore, we laid an additional constraint that the similarity measure should be *invariant to the form of the firing rate distribution*. These 3 constraints on metrics were essential to ensure that the metric was sensitive solely to the degree of similarity between the two spike trains, thereby enabling effective comparisons between input and output similarity.

To evaluate the robustness of the different similarity metrics to average firing rates of input patterns, we generated different sets of 11 morphed patterns (as illustrated in Figure 2), each with a different average firing rate value (5 Hz, 15 Hz, or 25 Hz). To assess invariance to population size, we generated different sets of 11 morphed patterns with 1000, 2000, or 3000 spike trains in each pattern. To examine the robustness of the similarity metrics to different firing rate distributions, we generated 2 sets of input patterns, one from a Poisson distribution and another from an exponential distribution, with average firing rate set at 8 Hz. For each of these 3 sets of input patterns, we computed the similarity between ${{P}_\beta }$ and ${{P}_0}$ for each of the 6 similarity metrics. We plotted the similarity measure values as functions of the morphing parameter $\beta $ for each of the 3 sets of patterns (Figure 2G-H; Supplementary Figure S4*E-H*).

Across all sets of patterns, all similarity metrics showed monotonic and gradual changes with reference to change in the morphing parameter. We found that Hamming distance, normalized Euclidean distance, cosine similarity, and mutual information measures were sensitive to changes in average firing rate and in firing rate distributions (Supplementary Figure S4*E-H*). In addition, the two distance-based metrics were sensitive to changes in population sizes as well (Supplementary Figure S4*E-F*). The correlation-based measures, ${{r}_\beta }$ and ${{R}_\beta }$, were invariant to all the 3 different tested criteria (Figure 2G-H). Based on these analyses, we chose ${{r}_\beta }$ and ${{R}_\beta }$ as similarity measures for comparing input and output patterns of our network.

### Sparse Firing and Pattern Separation in the Default DG Network Were Strongly Dependent on Interneuron Activity

**Figure 3.**
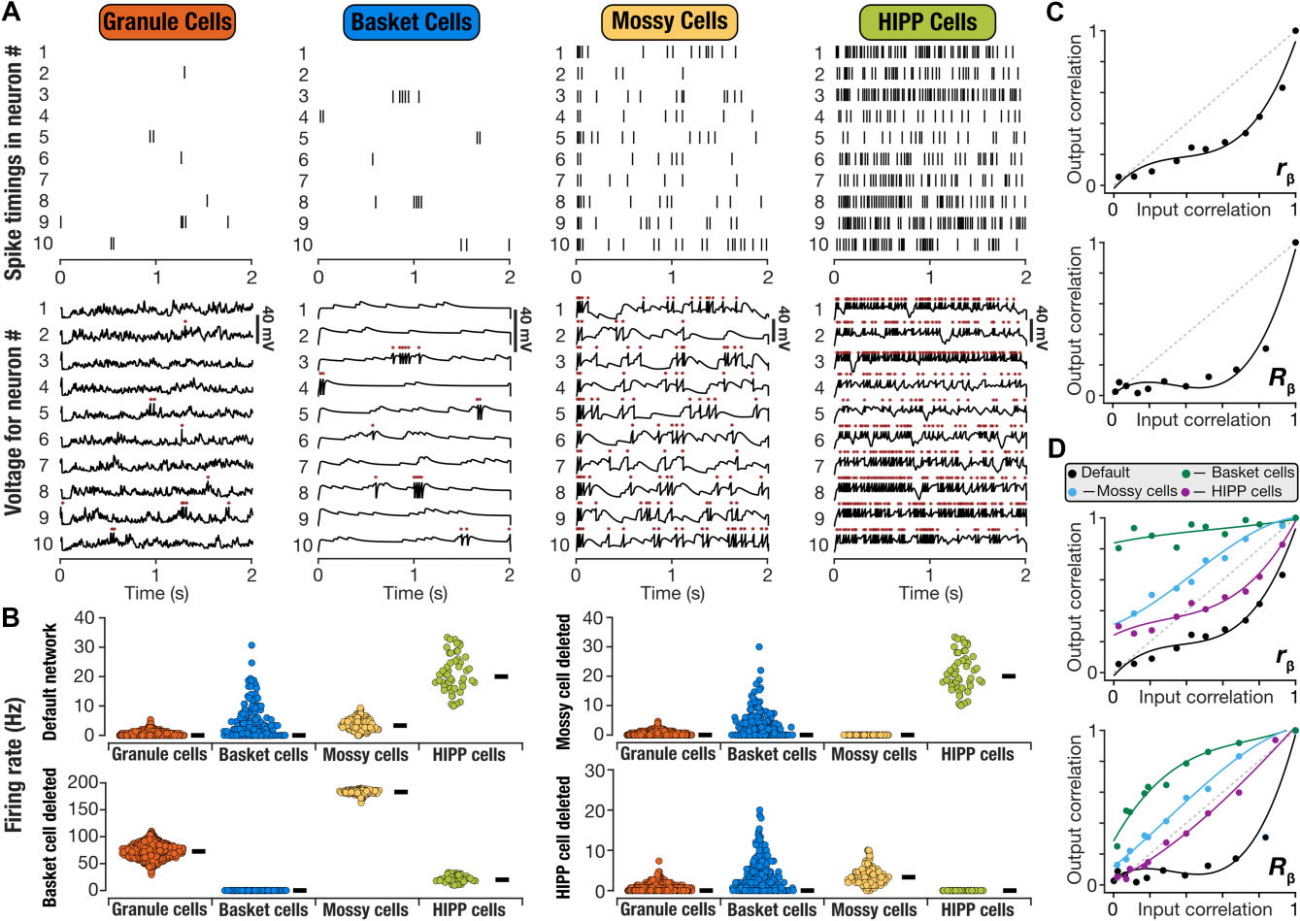
Deletion of different interneuron populations yielded differential impact on pattern separation performance of the default dentate gyrus network model. (A) Raster (*top*) and voltage traces (*bottom*) of 10 different granule, basket, mossy, and HIPP cells of the default DG network presented with the ${{P}_0}$ pattern. Asterisks above voltage traces indicate action potentials, which are represented by the raster plots above. (B) Distribution of firing rates for each neuronal subtype in the default network and in networks where each of the different interneuron subtypes (basket cells, mossy cells, and HIPP cells) were deleted. Supplementary Figure S6 provides representative raster plots of different neuronal subtypes from networks where each interneuron population was deleted. (C) The ability of the default network to perform pattern separation (output correlation < input correlation) represented using the plots of output correlation vs. input correlation, when correlation was measured using the ${{r}_\beta }$ (*top*) or ${{R}_\beta }$ (*bottom*) metrics. (D) Pattern separation in the default network and in networks where each of the different interneuron subtypes (basket cells, mossy cells, and HIPP cells) were deleted. Correlation was measured using either the ${{r}_\beta }$ (*top*) or ${{R}_\beta }$ (*bottom*) metrics. For panels C-D, the dots represent the input-output pairs when the network was presented with the 11 morphed patterns and the traces represent a cubic polynomial fitted to these data points.

We constructed a heterogeneous DG network by randomly picking non-repeating units of each neuron subtype from their respective valid model populations (Figure 1), with connectivity and synaptic parameters defined by the biological equivalents (Supplementary Tables S6-S7). We hand-tuned the synaptic weight parameters in this network such that ${{S}_{out}}$ was less than $\ {{S}_{in}}$ across input patterns, with similarity computed with either the ${{r}_\beta }$ or the ${{R}_\beta }$ metric. This yielded a heterogeneous DG network that executed pattern separation (Figure 3). In arriving at this network, we presented the 11 morphed inputs patterns to the network and computed the voltage responses of all neurons in the network to arrive at their spike patterns (Figure 3A). We computed the firing rates of all neurons in this default network and found neuron-to-neuron variability across all neuronal subtypes (Figure 3B).

We found granule cells to show low firing rates and sparse firing, with many cells eliciting no spikes during the 500-2000 ms period that was considered for analysis (Figure 3B). Quantitatively, we computed sparsity in granule cell firing as ($1 - {{f}_{{\mathrm{GC}}}}$), where ${{f}_{{\mathrm{GC}}}}$ defined the fraction of granule cells that elicited spikes. In the default hand-tuned network, granule cells showed sparse activity, with a sparsity value of 0.79. In contrast, the interneurons showed relatively low sparsity of action potential firing in the default network (BC: 0.58; MC: 0.02; HC: 0). We asked if sparsity in GC firing was simply consequent to the divergent connectivity of 700 PP inputs to 3600 granule cells, by computing firing rates in networks where individual interneuron subtypes were removed. We deleted individual interneuron subtypes from the network by removing all synapses to and from the specific subtype, while retaining the rest of the network to match the default network. We found that deletion of either basket cells or mossy cells markedly altered firing rates of the other neuronal subtypes, with the most dramatic differences observed with the removal of basket cells from the default network (Figure 3B; Supplementary Figure S5*B-C*). HIPP cells were unaffected by removal of interneurons, but removal of HIPP cells did alter firing of other cell types (Figure 3B; Supplementary Figure S5*D*). This was expected from the network architecture (Figure 1D) where HIPP cells did not receive inputs from other local neurons and project solely to the GCs (which is connected to the other 2 interneuron subtypes). Notably, consistent with previous lines of evidence on the roles of interneurons in GC sparsity,^12,17-20^ we found that removal of any of the interneuron subtypes from the default network resulted in reduction of GC sparsity (Supplementary Figure S5*B-D*; GC sparsity after bc removal: 0; GC sparsity after MC removal: 0.69; GC sparsity after HC removal: 0.71).

We used the average firing rates and spike patterns to compute the ${{r}_\beta }$ or the ${{R}_\beta }$ metric, respectively, for both the input and the output (action potentials from all GCs) patterns. We plotted output similarity vs. input similarity to confirm pattern separation in the default network, for the chosen set of ${{P}_0}$ and ${{P}_1}$ input patterns and their morphs (Figure 3C). To confirm that the hand-tuned DG network can perform pattern separation on different sets of randomly generated ${{P}_0}$–${{P}_1}$ patterns, we repeated our analyses with 10 additional sets of patterns. These sets of morphed patterns were presented to the same default network and pattern separation performance was assessed by plotting output vs. input correlations (Supplementary Figure S6*A*). We found that the default network manifested pattern separation with all these additional input sets as well (Supplementary Figure S6*A*). These results confirmed that the default network was not biased to the set of patterns used to hand-tune the network but could separate additional sets of input patterns as well.

In another set of simulations, we initialized our network with randomly re-picked heterogeneous neurons from their respective populations, with individual choices different from our original default network. For these simulations, we set the synaptic weights to be unchanged from our hand-tuned default network. We presented the morphed patterns, recorded the network outputs, computed both ${{r}_\beta }$ and ${{R}_\beta }$ across the input-output patterns of the networks (Supplementary Figure S6*B*). We found that pattern separation performance was dependent on the choice of specific neuronal populations as well, with certain initializations yielding comparable pattern separation performance while others hampering performance.

In these analyses, as GCs constitute the network output to the CA3, we computed output correlation from the action potentials of the GCs. Did the outputs of the other neuronal subtypes in the same default network manifest pattern separation with identical inputs? To answer this, we computed ${{r}_\beta }$ and ${{R}_\beta }\ $for the outputs of each interneuron subtype and plotted them against the respective input correlation values. We found that the outputs of basket cells and mossy cells showed pattern separation, but outputs of HIPP cells did not (Supplementary Figure S6*C*). As HIPP cells receive inputs solely from PP inputs (Figure 1D), the absence of pattern separation in these cell types was to be expected. In contrast, basket cells and mossy cells receive inputs from GCs (Figure 1D), implying that pattern separation in these cell types could be inherited from GCs. Alternately, as they send projections back to the GCs (Figure 1D), they could be playing a role in shaping pattern separation in GC outputs.

To assess the dimensionality of network activity and potentially visualize network response to different morphed patterns, we computed the principal components of the instantaneous firing rate vectors of all granule cells in the default network, obtained in response to all the 11 different input patterns (${{P}_0}$*–*${{P}_1}$). Consistent with the sparse granule cell firing in our pattern separating network (Figure 3A), the cumulative variance explained by the first 10 principal components was <40% (Supplementary Figure S6*D*). For the dominant 3 principal components, the cumulative explained variance was lower than 15%. Consequently, projections of activity patterns to a lower-dimensional space corresponding to the dominant 3 dimensions did not yield meaningful or interpretable trajectories, together emphasizing the sparse nature of activity patterns in granule cells (Supplementary Figure S6*D*).

To understand how different interneurons regulated pattern separation of GC outputs in the default network, we assessed pattern separation in the models where individual interneuron subtypes were deleted. We found that the GC outputs from networks that lacked any of the 3 interneuron subtypes were ineffective in executing pattern separation (Figure 3D), irrespective of whether pattern separation was assessed with the ${{r}_\beta }$ or the ${{R}_\beta }$ metric. The largest impact on the GC output correlations was observed when the basket cells were deleted (Figure 3D). Together, these observations demonstrated that sparse firing and pattern separation in DG granule cells were strongly regulated by the different DG interneuron subtypes.

### Multiple Pattern-Separating Networks Were Identified Through an Unbiased Stochastic Search Algorithm

Our hand-tuned default model offers one instance of the DG network that efficaciously implemented pattern separation. The choice of one single hand-tuned model offers a single solution that is biased by the parameters that were chosen for that network. Such biases also have been shown to reflect in how the network responds when challenged with perturbations such as deleting individual components.^54,66-73^ Therefore, we asked if the hand-tuned model was the only combination of synaptic weights in the DG network that could execute pattern separation. On the other extreme, is it possible that any random choice for these synaptic weight parameters yield efficacious pattern separation?

To address these questions, we modified the MPMOSS algorithm (Figure 1A) to search for valid network models, with the parametric space spanning the different synaptic weights (Supplementary Table S8) and the objective specified to be efficacious pattern separation by the network. We generated 20 000 different random DG networks by picking 8 synaptic weight values from their respective uniform distributions, without changing any of the other network properties. To each of these 20 000 networks, we presented all 11 input patterns (${{P}_0}$ to ${{P}_1}$) and obtained the outputs of the GCs for each pattern. We assessed the pattern separation capability of each network by plotting output correlation *vs*. input correlation, with correlation computed independently with the ${{r}_\beta }$ and ${{R}_\beta }$ metrics.

**Figure 4.**
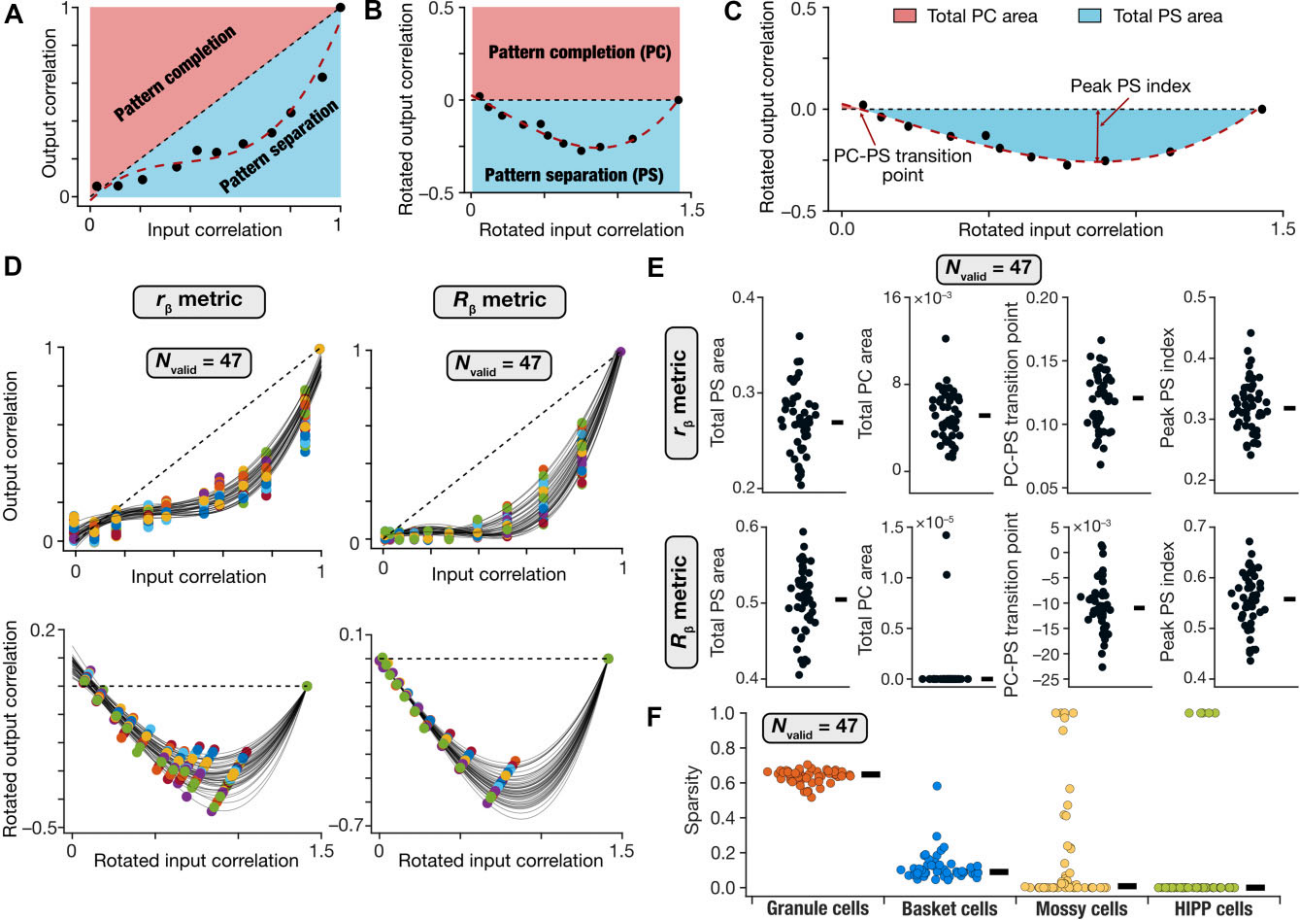
Unbiased search in the synaptic weights space yielded several DG networks that matched the performance criteria for pattern separation. (A) The ${{S}_{out}}$ vs. ${{S}_{in}}$ plot was derived from the inputs and outputs of the network using the ${{r}_\beta }$ and ${{R}_\beta }$ metrics. The ${{r}_\beta }$ plot for the default network is shown for this illustration. Note that pattern separation (PS) is defined to occur if ${{S}_{out}}$  *<*  ${{S}_{in}}$. Pattern completion (PC) occurs if ${{S}_{out}}$  *>*  ${{S}_{in}}$. (B) The data points from the plot in panel A were rotated clockwise by 45$^\circ $. A cubic polynomial was fitted to the rotated datapoints. In this rotated plane, pattern completion lies above the zero line, while pattern separation occurs below. (C) Quantitative measurements derived from the rotated plot of output vs. input correlation were used for validating models based on pattern separation performance. The plot and the fitted cubic polynomial shown are reproduced from panel B. Four measurements were defined using the fitted cubic polynomial: ${{A}_{PS}}$, the total pattern separation area; ${{A}_{PC}}$, the total pattern completion area; ${{T}_{\textit{PCPS}}}$, pattern completion to pattern separation transition point; and $P{{S}_{max}}$, the peak pattern separation index. Effective pattern separation was deemed to be achieved if ${{A}_{PS}} > 0.15$, ${{A}_{PC}} < 0.02$, ${{T}_{\textit{PCPS}}} < 0.25$, and $P{{S}_{max}}\ > \ 0.2$ (Supplementary Table S9). (D) Pattern separation plots for the 47 networks that satisfied all 4 measurement bounds computed with both ${{r}_\beta }$ and ${{R}_\beta }$ metrics. (E-F) Distributions of the 4 pattern separation measurements for ${{r}_\beta }$ (E; *top*) and ${{R}_\beta }$ (E; *bottom*) metrics as well as sparsity of different cell types (F) for all 47 valid DG networks.

Given the large number of randomly generated network models, it was essential to develop quantitative metrics for evaluating the pattern separation efficacy of each network toward their validation (Figure 4A-B). In doing this, we plotted ${{S}_{out}}$ vs. ${{S}_{in}}$ for all 11 patterns, independently with the ${{r}_\beta }$ or the ${{R}_\beta }$ correlation metric. For pattern separation to be realized, it was essential that ${{S}_{out}}$ be less than ${{S}_{in}}$ across the 9 intermediate morphed patterns (Figure 4A). With reference to the ${{S}_{out}}$  *vs*. ${{S}_{in}}$ plot, this translates to the need for all 9 intermediate points to fall below the 45-degrees (${{S}_{out}} = {{S}_{in}}$) equality line of the plot. To codify this requirement into our quantitative measurements, we first rotated the ${{S}_{out}}$ vs. ${{S}_{in}}$ plot by 45 degrees, with the 45-degree line forming the transformed $x$ axis (Figure 4B). In this rotated plane, the region above and below the zero-line represented pattern completion (PC) and pattern separation (PS), respectively (Figure 4B). We fitted a cubic polynomial to these rotated datapoints to arrive at quantifications that would maximize pattern separation and minimize pattern completion for our network outcomes.

We defined 4 quantitative measurements using the cubic polynomial fit to the ${{S}_{out}}$ vs. ${{S}_{in}}$ plot in the rotated plane (Figure 4C). We defined *total pattern separation area*, ${{A}_{PS}}$, as the total area under the curve of the negative-rectified cubic polynomial. *Total pattern completion area*, ${{A}_{PC}}$, was computed as the total area under the curve of the positive-rectified cubic polynomial. The point at which the fitted polynomial crossed zero was defined as the *PC-PS transition point*, ${{T}_{\textit{PCPS}}}$. The absolute value of the peak negative deflection of the fitted cubic polynomial represented the *peak pattern separation index*, $P{{S}_{{\mathrm{max}}}}$. With these quantitative definitions, effective pattern separation was achievable by maximizing ${{A}_{PS}}$, minimizing ${{A}_{PC}}$, minimizing ${{T}_{\textit{PCPS}}}$, and maximizing $P{{S}_{{\mathrm{max}}}}$ (Figure 4C; Supplementary Table S9).

We imposed bounds on each of these 4 measurements (Supplementary Table S9) for a network to be called a pattern separation network. We computed each of these measurements from the ${{S}_{out}}$ vs. ${{S}_{in}}$ plot spanning all 11 patterns for each of the 20 000 randomly generated networks, independently with the ${{r}_\beta }$ or the ${{R}_\beta }$ correlation metric. A network was declared to be a valid pattern separation network only if all 4 measurements (${{A}_{PS}}$, ${{A}_{PC}}$, ${{T}_{\textit{PCPS}}}$, and $P{{S}_{{\mathrm{max}}}}$) were within their respective bounds, with both ${{r}_\beta }$ and the ${{R}_\beta }$ correlation metrics. Thus, a total of 8 measurements (4 each with ${{r}_\beta }$ and ${{R}_\beta }$) were used for validating each network. The validation process yielded 47 valid DG networks, which was a small proportion of the generated random networks (∼0.2% of 20 000), that performed pattern separation (Figure 4D-E).

### Pattern-Separating Networks With Similar Granule Cell Activity Manifested Dissimilar Activity in the Interneurons

**Figure 5.**
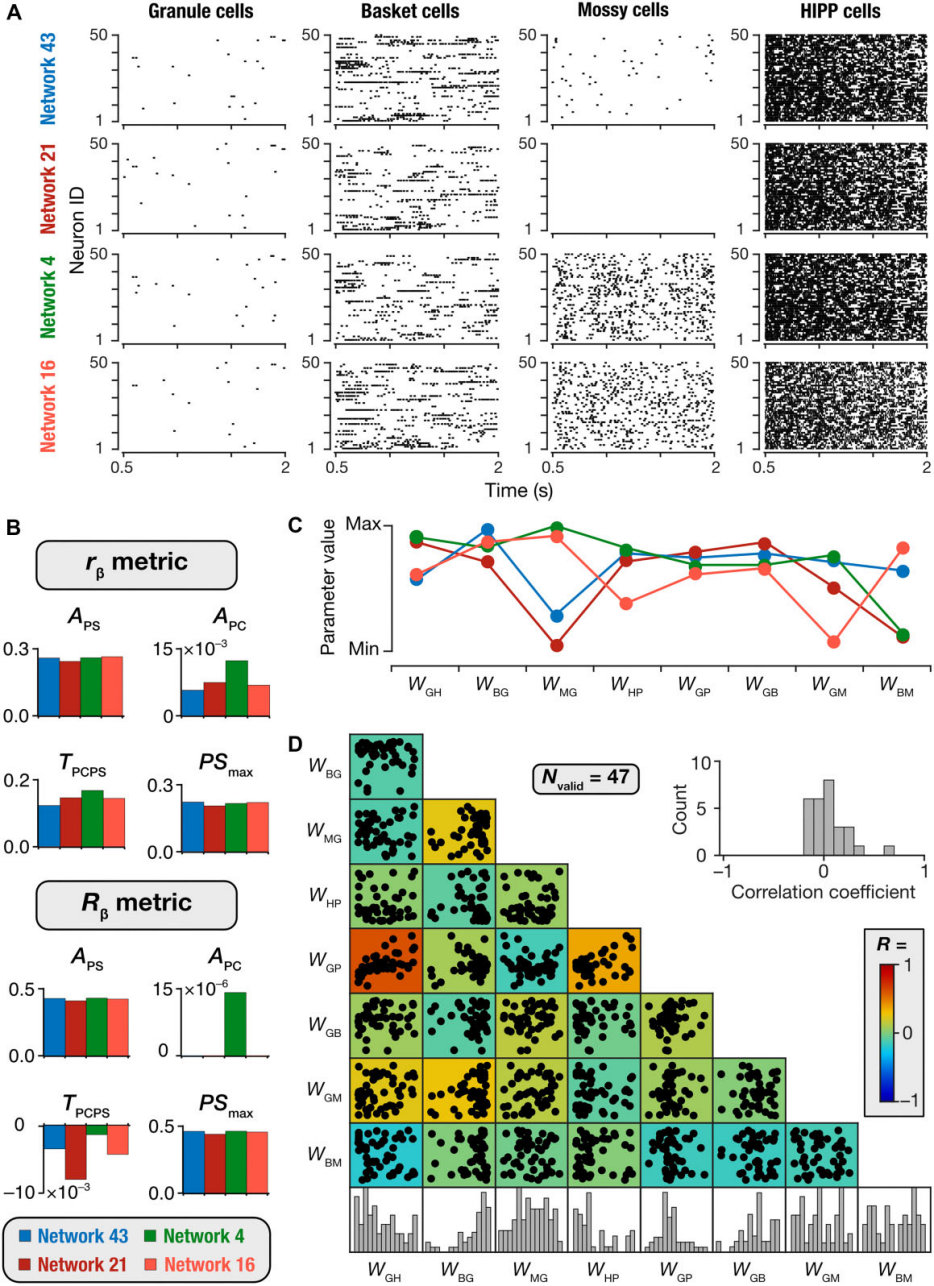
Networks with similar pattern separation performance and granule cell population activity manifested dissimilar activity across interneurons and pronounced variability in synaptic connectivity. (A) Raster plots showing firing of 50 granule (GC), basket (BC), mossy (MC), and HIPP cells (HC) of 4 selected networks where granule cell population activity was most correlated (computed through both ${{r}_\beta }$ and ${{R}_\beta }$ metrics for activity of all granule cells across networks). Although the granule cell population activity across the 4 networks were similar, the activity of interneurons manifested pronounced variability. Sparsity values for all neurons of each subtype in the networks also followed the same trend, showing similar sparsity for GCs but widely variable sparsity for the other interneurons. Network 43: GC: 0.63, BC: 0.05, MC: 0.41, HC: 0; Network 21: GC: 0.62, BC: 0.13, MC: 1, HC: 0; Network 4: GC: 0.62, BC: 0.1, MC: 0, HC: 0; Network 16: GC: 0.6, BC: 0.06, MC: 0, HC: 0; (B) Distributions of the 4 pattern separation measurements for ${{r}_\beta }$ (*top*) and ${{R}_\beta }$ (*bottom*) metrics for the 4 selected networks. (C) Distribution of the synaptic weights for 4 selected networks. (D) Pair-wise scatter plots and distributions of the 8 synaptic weights for all 47 valid DG networks. The color-coded heat map represents Pearson’s correlation coefficient values for each pairwise relationship. The inset depicts the distribution of all the 28 unique correlation coefficients in the scatter plot matrix.

How distinct were these 47 valid networks from each other in terms of the pattern separation measurements and the activity of the different neuronal subtypes? Although all valid networks satisfied all measurement bounds (for pattern separation) by requirement, we found marked network-to-network variability in their pattern separation (Figure 4D-E) and sparsity (Figure 4F) measurements. Importantly, although all 47 networks showed very similar sparsity values for their granule cells, the sparsity values for the different interneurons manifested pronounced network-to-network variability (Figure 4F). To explore this aspect of network-to-network variability, we first computed pairwise similarity between granule cell outputs of all 47 DG network using ${{r}_\beta }$ and ${{R}_\beta }$ measures. Using these pairwise similarity measures, we identified 4 networks with highest similarity in terms of granule cell outputs and sparsity values (Figure 5A). It may be noted that while the raster plots associated with 50 granule cells showed similar sparse firing across all 4 networks, action potential firing in mossy cells and basket cells were starkly different across the 4 networks (Figure 5A). With the network architecture (Figure 1D), HIPP cells did not receive any feedback from other neuronal subtypes in the network and therefore did not show marked variability across these 4 networks. Importantly, the pattern separation measurements of these 4 networks were also comparable, computed either using ${{r}_\beta }$ or ${{R}_\beta }$ measures (Figure 5B). These examples illustrate our observation that networks that had very similar granule cell activity and pattern separation performance were endowed with pronounced network-to-network variability in interneuron activity.

### Complexity and Degeneracy in Pattern-Separating Networks

How distinct were these 47 valid networks from each other in terms of the synaptic weights that defined them? To address this, we first considered the 4 networks that manifested similar granule cell activity (Figure 5A) and pattern separation performance (Figure 5B). We plotted the synaptic weights that connected the different neuronal subtypes within each of these 4 networks (Figure 5C). We found striking variability in the synaptic connectivity across these different networks, with even extreme scenarios where some networks lacked certain connections. For instance, in Network 21, mossy cells did not elicit action potentials (Figure 5A; mossy cell sparsity for Network 21 was 1) as there was weak connectivity from granule cells to mossy cells in this network (Figure 5C). Thus, despite strong similarities in DG activity (Figure 5A) and in pattern separation performance (Figure 5B) across 4 different networks, the underlying synaptic connectivity was extremely variable (Figure 5C).

To compare similarities of synaptic parameters across all 47 valid networks, we plotted the histograms of and pairwise correlations between the synaptic weight parameters governing these networks (Figure 5D). We found that the synaptic weight distributions spanned a large range of the search space (histograms in Figure 5D). Most synaptic weight parameters exhibited weak pairwise correlations (Figure 5D), except for relatively high correlation value between the excitatory PP-to-GC and inhibitory HC-to-GC weights, which was expected due to the network architecture (Figure 1D). Specifically, granule cells and HIPP cells receive common inputs from PP, and this strong correlation indicates the need to balance the excitation from the perforant pathway and the inhibition from the HIPP cells. Thus, valid pattern-separating networks did not cluster around a specific type of connectivity in achieving pattern separation through sparse firing across the granule cell population. Together, these results provide clear lines of evidence for synaptic degeneracy in heterogeneous DG network models, whereby disparate combinations of synaptic connectivity yielded similar pattern separation capabilities in DG networks.

### Network-to-Network Variability in the Impact of Deleting Individual Interneuron Subtypes on Pattern Separation

Our analyses showed that deletion of individual interneuron subtypes differentially hampered firing rates, sparsity, and pattern separation performance capabilities of granule cell outputs (Figure 3B, Figure 3D). These observations demonstrated that sparsity and pattern separation in DG networks are not solely dependent on the projection of low-dimensional PP inputs to a high-dimensional space involving a larger number of granule cells. There are several lines of evidence for important roles for the local DG network (Figure 1D) and the different interneuron subtypes in sparse firing and pattern separation.^12,17-20^ However, our observations on the differential roles of the individual neuronal subtypes were confined to the default network (Figure 3) and are prone to biases driven by connectivity patterns in the default network. Therefore, we extended our interneuron-deletion analyses to the 47 valid pattern-separating networks, especially considering the variability in synaptic connectivity and interneuron firing properties across these networks (Figure 4E, Figure 5).

**Figure 6.**
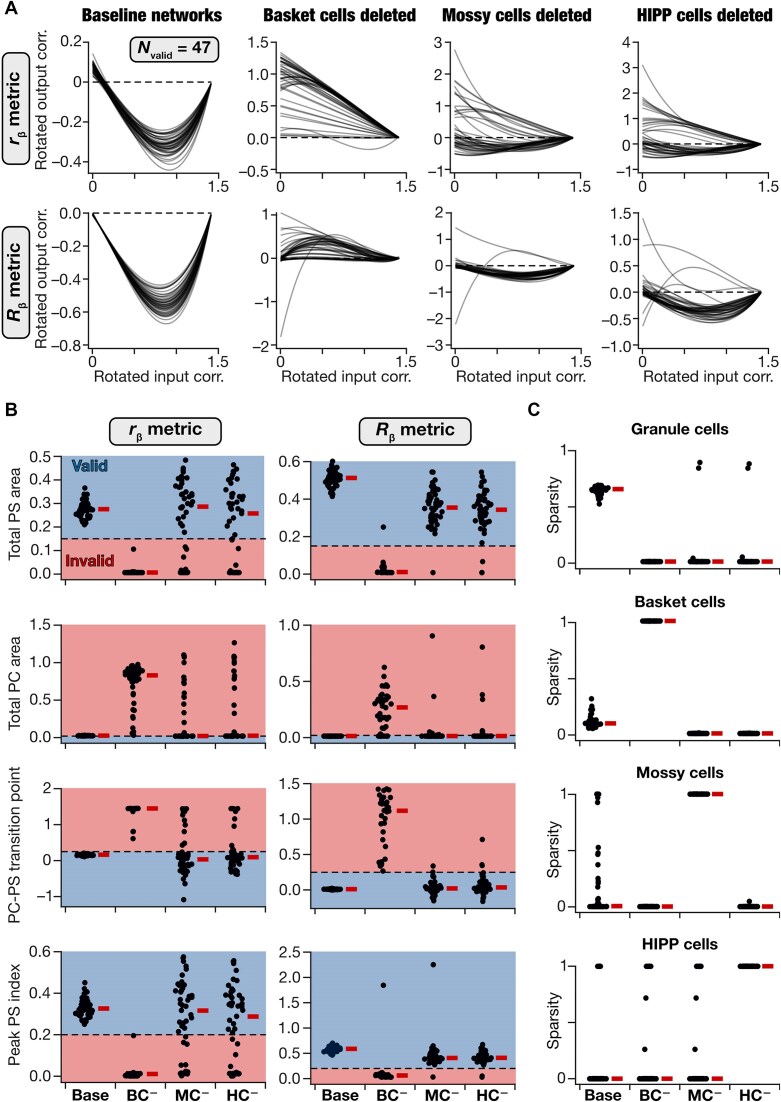
Pronounced network-to-network variability in the impact of removing individual interneuron subtypes on pattern separation performance and neural activity. (A) Visualization of pattern separation performance in all 47 valid baseline networks, and in each of the 47 networks with basket cells (BC), mossy cells (MC), or HIPP cells (HC) individually removed. Shown are fitted cubic polynomial on the rotated output correlation vs. rotated input correlation datapoints for all 11 morphed patterns. Correlation was computed either with the ${{r}_\beta }$ metric (*top*) or the ${{R}_\beta }$ metric (*bottom*). (B) All 4 pattern separation measurements in the valid networks and in networks where individual interneuron subtypes were deleted. These measurements were calculated from the traces shown in panel A for all 47 networks, with correlation computed either with ${{r}_\beta }$ (*left*) and ${{R}_\beta }$ (*right*). Blue and red shades indicate valid and invalid ranges for each measurement, respectively. (C) Sparsity of all neuronal subtypes in all 47 valid baseline networks and after deletion of individual interneuron subtypes. In (B-C), thick horizontal lines (placed on the right of the actual values) indicate the respective median values. Results of statistical analyses for these panels are presented in Supplementary Table S10.

We created a total of $3 \times 47 = 141$ networks that were altered by 3 different perturbations (where either all BCs, all MCs, or all HCs were deleted) in each of the 47 valid networks. We presented the 11 morphed patterns to each of these networks and computed sparsity of GC firing. We quantified pattern separation performance by assessing output correlations using ${{r}_\beta }$ and ${{R}_\beta }$ across the granule cells in each of the 141 networks (Figure 6A-B; Supplementary Table S10). First, we found that the impact of deletion on pattern separation was differential across different neuronal subtypes. Specifically, there was a striking loss of pattern separation performance across all networks when basket cells were deleted. However, deletion of either mossy or HIPP cells did not have as large an impact across networks (Figure 6A-B). Second, and more strikingly, there was pronounced network-to-network variability in how deletion of specific interneuron subtypes affected different networks. Whereas deletion of either mossy cells or HIPP cells had a large impact on pattern separation performance in certain networks, in other networks the impact was negligible (Figure 6A-B). In some of the networks, deletion of interneuron population even resulted in enhanced pattern separation capabilities, resulting in a diversity of network responses to deletions (Figure 6A-B). Remarkably, however, deletion of any of the 3 interneuron subtypes resulted in significant loss in sparsity of granule cells, basket cells, and mossy cells (Figure 6C; Supplementary Table S10).

Together, we demonstrate that the local DG circuit played a crucial role in regulating pattern separation across different networks. However, we report pronounced network-to-network variability in how interneurons affect pattern separation in the DG network, depending on the specific connectivity patterns in the network under consideration.

### Heterogeneous Networks Were More Robust to Synaptic Perturbations

The high proportion of random networks that were declared invalid (∼99.8%; Figure 5) demonstrates that pattern separation was highly sensitive to synaptic weight parameters. In biological networks, synaptic weights could undergo changes as the animals learn other tasks or undergo pathological changes. Heterogeneities in neural circuits have been proposed as a physiological mechanism to maintain resilience to perturbations in different kinds of neural circuits.^54,66-72,74^ Do heterogeneous DG networks maintain robust pattern separation in the face of synaptic perturbations? Would a homogeneous DG network maintain similar robust pattern separation in the presence of synaptic perturbations?

**Figure 7.**
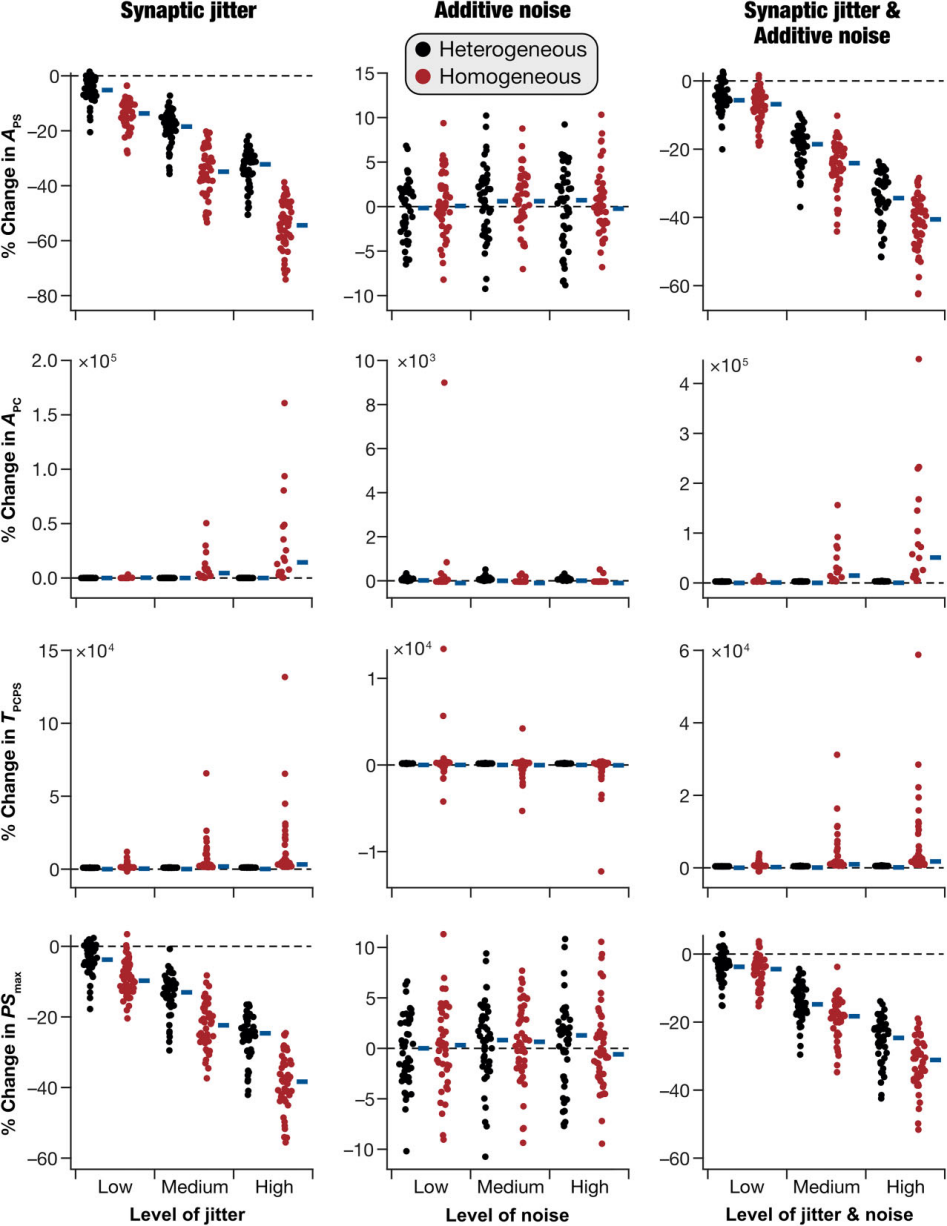
Heterogeneous dentate gyrus networks showed higher robustness in pattern separation performance when challenged with synaptic perturbations. Percentage changes in pattern separation measurements (computed using the ${{r}_\beta }$ metric; Supplementary Figure S13 provides measurements with the ${{R}_\beta }$ metric) when perturbations of different levels were introduced (with reference to respective base networks receiving no perturbations). Perturbations were introduced in 3 distinct levels (low, medium, and high) as synaptic jitter (*left column*), or as additive noise to synaptic currents (*middle column*) or a combination of both jitter and noise (*right column*). In all plots, thick horizontal lines (placed on the right of the actual values) indicate the respective median values. Results of statistical analyses for these panels are presented in Supplementary Table S11.

To address these questions, we compared the performance of heterogeneous and homogeneous dentate gyrus networks under different network perturbations. We introduced perturbations through jitter in synaptic weights and/or additive noise to synaptic currents. Both jitter and noise were Gaussian in nature, with increasing variances representing different graded levels. We assessed network’s pattern separation performance in all 47 homogeneous and 47 heterogeneous networks, each tested at different graded levels of jitter and/or noise (Figure 7, Supplementary Figures S7-S12). We computed the percentage change in all 8 pattern separation measurements (${{A}_{PS}}$, ${{A}_{PC}}$, ${{T}_{\textit{PCPS}}}$, and $P{{S}_{{\mathrm{max}}}}$ computed with ${{r}_\beta }$ and ${{R}_\beta }$ metrics) with reference to measurements from respective networks with no perturbations. We observed pronounced network-to-network variability, in both homogeneous and heterogeneous networks, in percentage changes of pattern separation measurements for the same levels of jitter and/or noise (Figure 7).

When these metrics were computed with ${{r}_\beta }$ for synaptic jitter (Figure 7; Supplementary Figures S7-S8), we found that ${{A}_{PS}}$ (Figure 7) and $P{{S}_{{\mathrm{max}}}}$ (Figure 7) showed significantly higher reduction (Supplementary Table S11) in homogeneous networks compared to their heterogeneous counterparts, indicating that heterogeneous networks were more robust to synaptic perturbations. Consistent with this, ${{A}_{PC}}$ (Figure 7) and ${{T}_{\textit{PCPS}}}$ (Figure 7) showed significant increases in homogeneous networks with synaptic jitter but did not change significantly in heterogeneous networks. These observations demonstrated that pattern separation performance of networks progressively degraded with graded increase in synaptic jitter, with heterogeneous networks more robust to jitter than their homogeneous counterparts. For the same set of networks, we found no significant difference in pattern separation performance between homogeneous and heterogeneous networks perturbed with synaptic jitter, when the ${{R}_\beta }$ metric was used for correlation computation (Supplementary Figure S13).

We repeated these analyses with additive noise (Figure 7; Supplementary Figures S9-S10) to synaptic currents as well as with the combination of jitter and noise (Figure 7; Supplementary Figures S11-S12). We did not observe significant changes in pattern separation performance (computed either with ${{r}_\beta }$ or ${{R}_\beta }$ metrics) of homogeneous vs. heterogeneous networks when additive noise was introduced into synaptic currents (Figure 7; Supplementary Figures S9-S10; Supplementary Table S11). We observed significant differences in robustness between heterogeneous and homogeneous networks when perturbations in both synaptic jitter and additive noise were introduced together, with the ${{r}_\beta }$ metric (Figure 7; Supplementary Figures S11-S12; Supplementary Table S11) but not with the ${{R}_\beta }$ metric (Supplementary Figures S11-S13).

## Discussion

How do we explain the several conflicting lines of evidence on the roles of different local-circuit interneurons of the dentate gyrus to pattern separation and sparsity? In addressing this question, we found that disparate combinations of non-random and non-unique synaptic connectivity yielded similar pattern separation efficacy in heterogeneous DG networks. These observations clearly explained the conflicting conclusions by unveiling synaptic degeneracy in the manifestation of pattern separation. The pronounced nature of network-to-network variability and the beneficial roles of heterogeneities in imparting resilience present a strong case for systematic characterization and analyses of the different forms of DG heterogeneities.

### Complexity and Multi-Scale Degeneracy in Heterogeneous Pattern-Separating DG Networks

Degeneracy, the ability of disparate structural components to yield similar functional outcomes, is a ubiquitous feature of biological systems across all scales of analysis.^66,73,75-80^ Our analyses demonstrate the manifestation of multi-scale degeneracy in pattern separating DG networks. At the cellular scale, consistent with prior studies from different DG neuronal subtypes,^26,34,54-57^ we found that disparate parametric combinations yielded signature functional characteristics of individual neurons belonging to 4 different subtypes (Figure 1; Supplementary Figure S1). At the network scale, several non-unique combinations of synaptic weight combinations yielded pattern separating DG networks with sparse firing (Figures 4, 5). The concurrent expression of cellular and network-scale degeneracy emphasizes the inherent flexibility and adaptability of the DG network,^26,54,81,82^ with several degrees of freedom available toward achieving pattern separation.

The manifestation of heterogeneities and degeneracy rules out the presence of a unique, completely determined solution that yields functionally precise neuronal models (at the cellular scale) or a pattern-separating DG network (at the network scale). In addition, we found large proportions of randomly generated neuronal models (Figure 1B) or network models (∼99.8%; 19 953 of 20 000) to be invalid. Therefore, in both cellular and network scales, arbitrarily random combinations could not yield collective functional outcomes. These observations provide direct evidence that pattern-separating DG networks are neither fully determined nor are completely random. They require specific non-random interactions among components to yield collective neuronal or circuit function. The manifestation of such intermediate levels of randomness along with the expression of degeneracy indicate that pattern-separating networks are complex systems.^75,83-87^

Complex systems execute their collective function through non-unique and non-random interactions among their functionally specialized subsystems. The ability of disparate functional subsystems to yield similar collective function through non-random, motif-based interactions is a hallmark of complex systems. Thus, our analyses argue for approaching pattern-separating DG networks from the complex systems framework. This implies that the relationship between structural components and collective functional outcomes is not one-to-one, but many-to-many.

### Multiple Subsystems Differentially Contribute to Pattern Separation in Heterogeneous DG Networks

Our observations with the population-of-networks approach are in striking contrast with the classical Marr-Albus theory, where pattern separation is postulated to be realized by divergent feedforward excitation.^1,2^ First, despite being endowed with the same divergent afferent connectivity and despite being built of the same set of neurons, we found that pattern separation couldn't be achieved in a vast majority of networks where local connectivity was randomized (Figures 4-5). These observations demonstrate that divergent connectivity and low excitability of granule cells are not sufficient to guarantee pattern separation. Instead, consistent with other observations from the DG network,^12,17-20^ our results suggest a critical role for local interneurons in regulating pattern separation and sparse firing. These conclusions are further validated by the strong dependency of pattern separation on local synaptic weights. Specifically, we showed that a majority of randomly connected networks were invalid (Figures 4-5) and deletion of interneurons (Figure 6) or synaptic jitter (Figure 7) degraded pattern separation performance of the networks.

Second, within the pattern-separating networks, we found pronounced network-to-network variability in firing rates and sparsity of interneuron populations, in local synaptic weights, and in how different interneuron populations contributed to pattern separation and sparsity (Figures 4-5). This is consistent with the manifestation of degeneracy, where different neural circuits performing the same function (through different combinations of subsystems) show differential dependencies on individual subsystems.^54,66-72^ More importantly, the complex system framework and network-to-network variability provide perfect substrates for explaining discrepancies in the literature on interneuron contributions. Specifically, the several recurrent connections between excitatory and inhibitory interneurons in the DG network have yielded puzzling conclusions about the role of each neuronal subtype in regulating sparsity and pattern separation.^8,10-12,14,16-20,36-42^ Our observations show that there are several non-random synaptic connectivity patterns that can yield effective pattern separation, with pronounced network-to-network variability in the roles of interneuron subtypes.

Future studies should approach pattern separation in the DG network not exclusively from the perspective of one-to-one relationship between individual components and function. Instead, the focus must be on the global structure associated with how different components interact with each other in several non-unique, non-random ways to yield pattern separation. Such analyses should account for the manifestation of network-to-network variability in dependencies on specific components, ensuring that the heterogeneities across networks are respected and all networks are not lumped into one homogeneous population. It is equally important to account for heterogeneities in the proportions of neuronal subtypes in different parts of the dentate gyrus.^25^

Our conclusions on degeneracy, complexity, and network-to-network variability were possible only because we used a population-of-networks approach to study the DG network. The choice of one hand-tuned model offers a single solution that is biased by the parameters that were chosen for that network. Such biases have been demonstrated to reflect in how the network responds when challenged with perturbations such as deleting a component.^51,54,69,71,72,88-94^ Together, our analyses strongly advocate the use of a population-of-networks approach, involving networks with different heterogeneities, in assessing pattern separation in DG networks. The complex systems framework and the population-of-networks approach together offer an ideal route to account for the several DG circuit components and heterogeneities therein, toward implementing robust pattern separation through flexible mechanistic routes.

### Heterogeneities, Metrics for Circuit Performance, and Robustness to Perturbations

Biological systems are characterized by their robust execution of function despite the expression of perturbations. There are lines of evidence that the manifestation of heterogeneities could offer a biological mechanism to yield robustness in certain types of networks.^54,66-72,74^ Our analyses also demonstrate that DG networks endowed with intra-subtype heterogeneities were more robust to the synaptic weight perturbations compared to their homogeneous counterparts (Figure 7). However, our analyses also highlight the need for the use of different metrics in assessing circuit function. Specifically, our analyses demonstrated that pattern separation performance computed from correlations of average firing rates (${{r}_\beta }$) showed progressive degradation with increasing level of jitter in synaptic weights, but not with additive noise. These analyses also indicated that heterogeneous networks were more resilient to synaptic jitter compared to their homogeneous counterparts (Figure 7).

In striking contrast, when we computed pattern separation performance through correlations across spike trains (${{R}_\beta }$) instead of average firing rates, pattern separation performance did not manifest large differences and there were no differences between heterogeneous and homogeneous networks as well (Supplementary Figure S13). Specifically, ${{r}_\beta }$ and ${{R}_\beta }$ could be considered to be different in terms of smoothing time windows and the specific representation (scalar vs. vector) of firing associated with each neuron. The metric ${{r}_\beta }$ uses a larger time window in computing the average firing rate across the entire time duration. This yields a single scalar (firing rate) that represents single-neuron firing, and thus does not account for temporal pattern of the spikes. On the other hand, in computing ${{R}_\beta }$ (Supplementary Figure S3), we used a low-variance, smaller timescale Gaussian kernel to smooth the spike trains. This results in a vector along the time axis as the representation of firing activity in each neuron, thereby allowing retention of temporal information of where the spikes specifically occurred. Thus, the temporal pattern of spikes also plays a role in determining ${{R}_\beta }$. Together, our analyses provide lines of evidence that the additional information carried by the temporal pattern of spikes might offer efficacious pattern separation despite perturbations.^95,96^ In this context, it is important to note that the specific value of ${{R}_\beta }$ would be influenced by the variance of the Gaussian kernel used to compute instantaneous firing rates^26^, together allowing a continuum of correlation metrics that spans both ${{R}_\beta }$ (small timescale) and ${{r}_\beta }$ (large timescale spanning the entire window). These observations also emphasize the need to use multiple metrics to assess network performance, to avoid biases associated with the use of a single metric in evaluating circuit function.

### Future Directions Within the Complex Adaptive Systems Framework

The complex adaptive systems perspective for the emergence of circuit function provides a unified framework to understand heterogeneities and how they contribute to collective function of the network and robustness therein. As our analyses showed, the focus here must be on how different forms of heterogeneities (eg, in neuronal intrinsic properties, local synaptic connectivity, sparsity of afferent connectivity, and neuronal morphology) interact with each other toward achieving collective function and resilience therein. Context-dependent multi-component plasticity during behavioral learning provides the means to align different forms of heterogeneities toward yielding robust function.^5,77,97-101^ Such multi-component plasticity and its specificity to behavioral contexts could be mediated by temporally aligned adult neurogenesis, which offers a hyperplastic substrate to regulate sparsity of representation by implementing plasticity heterogeneity.^9,12,26,28-35,54^ Structured multi-component plasticity driven by behavioral context, with adult neurogenesis as the substrate, offers a flexible set of adaptive mechanisms that recruit different circuit components to yield one of several robust solutions for sparse firing and pattern separation in DG circuits.^26,54,77,81,82^

Thus, the complex adaptive systems framework unifies disparate lines of research on the dentate gyrus on different forms of plasticity, adult neurogenesis, engram formation, and resource allocation toward selection of specific combinations of heterogeneities that yield robust circuit function.^9,12,26,28,30,31,33,34,44,77,81,100,102-105^ Importantly, findings on representational drift in network representations for the same behavioral context^106-109^ could be effectively fit within the complex adaptive systems framework, as representational drift simply implies that the complex network is traversing across different degenerate solutions that retain functional precision.

Future computational models could focus on *adaptive emergence of* effective pattern separating networks through multi-component plasticity in random networks that are endowed with different cell types, plasticity in neuronal properties and connectivity, and hyperplasticity in a subset of adult-born neurons. In pattern separating networks arrived through such an *adaptive learning* process, the manifestation of specific network motifs^110^ that resemble connectivity in the DG microcircuit could be assessed. The specific roles of intrinsic and synaptic plasticity (across different cell types) in the emergence of pattern separating networks and underlying motifs could then be assessed within the complex adaptive systems framework. Heterogeneities across different neuronal populations and their connectivity, the contributions of temporally-aligned adult neurogenesis in encoding specific contexts, representational drift, and network-to-network variability in the role of different interneurons could be easily explained within this framework.

Experimentally, it is important that studies that assess pattern separation systematically account for the different forms of heterogeneities that the DG is endowed with as well as animal-to-animal variability in different components that contribute to pattern separation. Importantly, analyses of neurological disorders affecting hippocampal function should consider different routes to dysfunction, including possibilities where unstructured changes to heterogeneities could result in loss of collective functions under pathological conditions.^74,77,111^ Together, the complex adaptive systems framework offers a unified framework for tying together the disparate lines of research on the heterogeneous dentate gyrus network and could be effectively harnessed for studying its multifarious functions under both health and disease conditions.

## Materials and Methods

Neural circuits are heterogeneous. Heterogeneities span structural characteristics, intrinsic properties, local synaptic connectivity, and afferent connectivity onto individual neurons in the network. A fundamental question in neuroscience is to understand the origins, the prevalence, and the implications of these heterogeneities to neural circuit physiology. Although the pronounced manifestation of heterogeneities in different cellular-scale properties is well-characterized in the dentate gyrus (DG), the implications for these heterogeneities to its circuit-scale function have not been thoroughly assessed. Our goal here was to study the implications of different neural heterogeneities on pattern separation in the hippocampal dentate gyrus network. In addressing this, we took a population-of-models approach that involved a multi-scale cascade of unbiased stochastic searches. Specifically, 4 independent stochastic searches were first used to find physiologically validated heterogeneous cellular-scale models of 4 different neuronal subtypes. A second level of unbiased stochastic search was performed to identify networks composed of these validated neuronal models that were structurally constrained by the DG architecture and functionally validated for their ability to perform pattern separation. We used the population of networks that emerged from the second search to assess heterogeneities and degeneracy in these networks toward the emergence of pattern separation capabilities.

### Subtype-Specific Single-Neuron Models

We first ensured that the constituent single-neuron model populations functionally matched the DG neuronal subtypes and their specific heterogeneities. The DG network was constructed with 4 neuronal subtypes: the principal granule cells and 3 different interneuron subtypes, namely basket cells, mossy cells, and HIPP cells. Irrespective of neuronal subtype, we modeled individual cells using the adaptive exponential integrate-and-fire (aEIF) spiking neuronal model^112^ given their ability to match different physiological characteristics and the low computational complexity. The choice of the aEIF model for implementing all subtypes allowed for exploration of intra- and inter-subtypes heterogeneities within the same parametric and measurement spaces.

The membrane potential dynamics of aEIF neurons was defined by the following system of ordinary differential equations^112^:


(1) \begin{eqnarray*}
{{c}_m}\frac{{d{{V}_m}}}{{dt}} = \ {{g}_l}\left( {{{E}_l} - {{V}_m}} \right) + {{g}_l}{{\Delta }_T}\exp \left( {\frac{{{{V}_m} - {{V}_{th}}}}{{{{\Delta }_T}}}} \right) + \sum {{I}_{syn}} - w,
\end{eqnarray*}

where ${{V}_m}$ represented the membrane potential (in ${\mathrm{mV}}$), ${{c}_m}$ and ${{g}_l}$ defined the membrane capacitance (in ${\mathrm{\mu F}}$) and the leak conductance (in ${\mathrm{mS}}$), respectively. ${{E}_l}$ was the reversal potential for the leak conductance (in ${\mathrm{mV}}$), ${{{\mathrm{\Delta }}}_T}$ was the slope factor (in ${\mathrm{mV}}$), ${{V}_{th}}$ was the threshold (in ${\mathrm{mV}}$), ${{I}_{syn}}$ was the synaptic current (in ${\mathrm{nA}}$). The dynamics of the adaptation parameter $w$ in eqn (1) evolved as


(2) \begin{eqnarray*}
{{\tau }_w}\frac{{dw}}{{dt}} = \alpha \left( {{{V}_m} - {{V}_{th}}} \right) - w,
\end{eqnarray*}

where ${{\tau }_w}$ defined the time constant association with $w$ and $\alpha $ was the adaptive coupling parameter. We used surface area ($A$) as representation for the structural properties of the neurons, which was used to compute ${{c}_m}$ and ${{g}_l}$ in eqn (1) as follows:


(3) \begin{eqnarray*}
{{c}_m} = {{C}_m} \times A
\end{eqnarray*}

(4) \begin{eqnarray*}
{{g}_l} = \frac{A}{{{{R}_m}}},
\end{eqnarray*}

where ${{C}_m}$ and ${{R}_m}$ represented the specific membrane capacitance (in ${\mathrm{\mu F}}/{\mathrm{c}}{{{\mathrm{m}}}^2}$) and specific membrane resistance (in ${\mathrm{k\Omega }} \cdot {\mathrm{c}}{{{\mathrm{m}}}^2}$), respectively.

As the dynamics evolved for different synaptic inputs, the neuron was considered to have elicited an action potential every time the value of ${{V}_m}$ [computed from eqn (1)] crossed the threshold voltage ${{V}_{th}}$. The occurrence of a spike also triggered updates in the values of ${{V}_m}$ and $w$ as follows:


(5) \begin{eqnarray*}
\begin{array}{@{}*{1}{l}@{}} {{\mathrm{At}}\;t = {{t}_{\textit{spike}}}{\mathrm{\ }}\left( {{{V}_m} \ge {{V}_{th}}} \right)}\\ {{\mathrm{Reset}}:\;V \to {{V}_{\textit{reset}}};w \to w + b} \end{array}
\end{eqnarray*}

where $b\ $ defined the spike-triggered adaptation parameter and ${{V}_{\textit{reset}}}$ denoted the voltage to which ${{V}_m}$ was reset after a spike. A refractory period of 5 ms was imposed following every spike, during which no further spiking was allowed.

The default values of the 10 parameters that defined models for each of the 4 cell types are listed in Supplementary Tables S1-S4.

### Intrinsic Electrophysiological Properties of the 4 Neuronal Subtypes

We validated models for each neuronal subtype against 6 electrophysiological measurements: membrane time constant $( {{{\tau }_m}} )$, sag ratio (Sag), input resistance$\ ( {{{R}_{in}}} )$, spike frequency adaptation $( {SFA} ),$ and action potential firing frequencies for 50 pA and 150 pA pulse current injections $({{f}_{50}}$ and ${{f}_{150}}$, respectively$)$. All measurements were obtained after an initial delay period of 500 ms, during which the transient membrane dynamics of the model stabilized to its resting potential. These measurements were computed using established protocols for computing each of them^23,26,113-115^ and are elaborated below.

The membrane time constant $( {{{\tau }_m}} )$ was estimated by fitting a single exponential to the membrane potential response to a 20-pA pulse current injected into the neuron for 1500 ms:


(6) \begin{eqnarray*}
{{V}_m} = {{V}_{\infty \ }}\left( {1 - \exp \left( { - \frac{t}{{{{\tau }_m}}}} \right)} \right) + {{V}_0},
\end{eqnarray*}

where ${{V}_\infty }$ denoted the steady-state voltage response and ${{V}_0}\ $ represented the resting potential at which the pulse current was injected.

Sag ratio was measured as the ratio of the steady-state voltage deflection $({{V}_{SS}})$ to the peak voltage deflection (${{V}_{\textit{peak}}}$) obtained in response to −50 pA current pulse for one second. Input resistance $( {{{R}_{in}}} )$ was calculated by injecting a family of nine 1-s-long pulse currents with amplitudes ranging from −40 pA to 40 pA in steps of 10 pA. Steady-state voltage deflections were calculated for each pulse current injection. ${{R}_{in}}$ was then computed as the slope of the steady-state voltage deflection *vs*. current injected plot for all 9 pulse current amplitudes. As HIPP cells elicit action potentials for current amplitudes above 20 pA, we used step currents ranging from −20 pA to 20 pA in steps of 5 pA for ${{R}_{in}}$ computation in HIPP cells. Firing frequencies (${{f}_{50}}$ and ${{f}_{150}}$) were computed as the number of spikes elicited by the neuron upon injection of 50 pA and 150 pA pulse currents, respectively, for 1 s duration each. Spike frequency adaptation (SFA) was measured as the ratio of the first inter-spike interval to the last inter-spike interval for a pulse current of 150 pA amplitude injected for 1 s. The electrophysiological ranges for all 6 measurements derived from previous electrophysiological studies^23,26,113-115^ are listed in Supplementary Table S5.

### Generation of Heterogeneous Populations of Functionally Validated Models for the 4 Neuronal Subtypes

We needed physiologically validated heterogeneous neurons of all 4 neuronal subtypes to construct the DG network. We used 4 independent implementations of MPMOSS algorithm (Figure 1A) to generate heterogeneous model populations of all 4 neuronal subtypes. The MPMOSS algorithm has been widely used to study the emergence of signature physiological characteristics in neurons and their networks through disparate combinations of different parameters,^48-53,116^ including in DG neurons with different model complexities.^26,34,54-56^ We chose an unbiased random search approach covering a large swath of the parametric space, rather than confining search spaces to constrictive subspaces, to allow for model heterogeneities to potentially span the entire parametric space.

In implementing MPMOSS, we first generated one valid model for each neuronal subtype by hand-tuning the parameters such that all 6 measurements were in their respective valid range (Supplementary Table S5). These models then served as the substrate for performing the stochastic search where we specified upper and lower bounds for each parameter around the parameters of the default network (Supplementary Tables S1-S4). To ensure that the search remained unbiased to any specific distribution, these bounds were used to define uniform distributions for each parameter for each neuronal subtype. Each parameter was randomly sampled several times ($N$ = 100 000 for granule cells, 60 000 for basket cells, 10 000 for mossy cells, and 20 000 for HIPP cells) from its respective distribution, together creating several multi-parametric random models of each neuronal subtype (Figure 1A).

All 6 electrophysiological measurements were computed for each of these random models and were independently validated against the electrophysiological measurement ranges of the respective neuronal subtype (Supplementary Table S5). A model was declared valid only if all measurements were within their respective physiological ranges; otherwise, the model was considered invalid. This validation process, applied to every generated model across the 4 neuronal subtypes, yielded a subset of models that were valid (${{N}_{\textit{valid}}}$) for each neuronal subtype. The heterogeneities within and across valid models of different neuronal subtypes, in the 9-dimensional parametric space and the 6-dimensional measurement space, were visualized using principal component analysis. In addition, statistical analyses involving the histogram of individual parameters and pairwise correlations between valid model parameters/measurements were performed for each of the 4 neuronal subtypes to assess heterogeneities.

### Dentate Gyrus Network Model Endowed With Within-Subtype Heterogeneities

Valid models of the 4 neuronal subtypes were then used to construct the heterogeneous DG network with non-repeating units. We built a network made of 3600 granule cells, 500 basket cells, 180 mossy cells, and 50 HIPP cells (Figure 1D). The relative proportions of each cell type were informed by previous studies.^26,37,45,47^ The primary afferent input to the DG is the perforant pathway (PP), which we modeled using 700 independent spike trains, with specific firing rates and spike timing distributions. Granule cells functioned as the primary output neurons of the DG, projecting excitatory synapses onto CA3 pyramidal cells.

The connectivity within the DG circuitry (Figure 1D) was adopted from previous work.^37^ Specifically, HIPP cells, as GABAergic interneurons, established inhibitory synapses onto granule cells. Granule cells made excitatory synaptic connections with mossy cells and basket cells. Mossy cells, which are glutamatergic interneurons, connected to both granule and basket cells through excitatory synapses. Basket cells, which are inhibitory interneurons, established inhibitory connections with granule cells. The afferent inputs (PP) and all the neuronal subtypes together formed 8 types of synapses in the model. The specific connectivity between individual neurons or with afferent inputs was established randomly, by specifically accounting for the different connection probabilities (Supplementary Table S6) derived from an earlier study.^37^ For the default network, the weights of all connections were hand-tuned toward achieving effective pattern separation by the DG network (see below).

### Modeling Synapses

Given the nature of the individual neuronal subtypes that were used to construct the network, there were excitatory (glutamatergic) and inhibitory (GABAergic) synapses that connected different neurons in the network. The excitatory synapses were modeled as synaptic current mediated through colocalized AMPA and NMDA receptors, which were activated upon spiking of the respective presynaptic neuron. The inhibitory synapses were modeled as current mediated through GABA_A_ receptors. The currents through AMPA and GABA_A_ receptors were modeled using the following formulation:


(7) \begin{eqnarray*}
{{I}_{syn}} = {{g}_{syn}}\left( t \right)\left[ {{{V}_m}\left( t \right) - {{E}_{syn}}} \right]
\end{eqnarray*}

(8) \begin{eqnarray*}
{{g}_{syn}}\left( t \right) = {{\bar{g}}_{syn\ }}u\left( t \right),
\end{eqnarray*}

where ${{I}_{syn}}$ represented the synaptic current, ${{E}_{syn}}$ denoted the reversal potential of the receptor ($0\ {\mathrm{mV}}\ $for AMPA and NMDA receptors and $ - 90\ {\mathrm{mV}}$ for GABA_A_ receptors), ${{V}_m}$ defined the membrane potential and ${{g}_{syn}}( t )$ tracked the evolution of receptor conductance. ${{g}_{syn}}( t )$ was computed as a scaler multiple of $u( t )$, a double exponential waveform to represent the rise and decay of synaptic currents.


(9) \begin{eqnarray*}
u\left( t \right) = \exp \left( { - \frac{t}{{{{\tau }_{\textit{decay}}}}}} \right) - \exp \left( { - \frac{t}{{{{\tau }_{\textit{rise}}}}}} \right),
\end{eqnarray*}

where ${{\tau }_{\textit{rise}}}$ and ${{\tau }_{\textit{decay}}}$ defined the time constants associated with the rise and decay of synaptic conductance, respectively. The values for ${{\tau }_{\textit{rise}}}$ and ${{\tau }_{\textit{decay}}}$ for the receptors associated with the different synapses in the network (Supplementary Table S7) were taken from previous studies.^23,37,47,58,59^  ${{\bar{g}}_{syn\ }}$defined the peak conductance of the AMPA and GABA_A_ receptors and will be referred to as the weight of the connection.

The dependence of the current through NMDA receptor on postsynaptic membrane voltage was modeled as


(10) \begin{eqnarray*}
{{I}_{syn}} = {{g}_{syn}}\left( t \right)\ s\left( V \right)\ \left[ {{{V}_m}\left( t \right) - {{E}_{syn}}} \right]
\end{eqnarray*}

(11) \begin{eqnarray*}
s\left( V \right) = \frac{1}{{1 + \eta \ {{{\left[ {{\mathrm{M}}{{{\mathrm{g}}}^{ + + }}} \right]}}_o}\ exp\,\left( { - \gamma {{V}_m}} \right)}},
\end{eqnarray*}

where $s( V )$ defined the additional magnesium-dependent mechanism of the NMDA receptors. The parameters that defined the sigmoid, $\eta \ $was set at$\ 0.28\ {\mathrm{m}}{{{\mathrm{M}}}^{ - 1}}$, ${{[ {M{{g}^{ + + }}} ]}_o}$ was $1\ {\mathrm{mM}}$, and $\gamma $ was$\ 0.04\ {\mathrm{m}}{{{\mathrm{V}}}^{ - 1}}$. Synaptic conductance ${{g}_{syn}}( t )$ for NMDARs followed a similar formulation as in eqns (8)-(9). ${{\bar{g}}_{{\mathrm{syn}}\ }}$of NMDA receptors was defined as a scaled version of the ${{\bar{g}}_{{\mathrm{syn}}\ }}$of AMPA receptors (Supplementary Table S7), with a fixed NMDAR: AMPAR ratio used for scaling.^37^

We introduced delays between presynaptic spike and the onset of the corresponding postsynaptic responses. Different values for delays were used for different connections (Supplementary Table S7), which were taken from previous studies.^23,47,58,59^

### Modeling Perforant Pathway Inputs

Our model contained 700 PP inputs impinging on the granule and HIPP cells. We modeled 700 PP inputs as 700 independent spike trains generated from a Poisson distribution with $\lambda $ = 8 Hz (Figure 2A) and inter-spike interval drawn from a corresponding exponential distribution (Figure 2B). We generated 2 distinct input patterns ${{P}_0}$ and ${{P}_1}$ by randomly generating 700 spike trains for each pattern. These 2 patterns formed the extreme inputs that were fed as inputs to the DG network. We confirmed the distinctiveness of these two patterns by ensuring that the input correlation measures computed between the 2 patterns approached zero. To assess pattern separation capabilities of the network, we used the morphed input approach where ${{P}_0}$ was progressively morphed into ${{P}_1}$ through several intermediate input patterns.^3,60,81,82^ Specifically, we generated 9 more intermediate input patterns by progressively morphing ${{P}_0}$ to ${{P}_1}$, yielding a total of 11 input patterns ${{P}_0},\ {{P}_{0.1}},\ {{P}_{0.2}},.\ .\ .,\ {{P}_{0.9}}\ $ and ${{P}_1}$, referred to as ${{P}_\beta }$ with $\beta $ representing the morphing parameter. With increasing $\beta $, patterns progressively became more dissimilar to ${{P}_0}$ and more alike ${{P}_1}$. For generating pattern ${{P}_\beta }$, $\beta $ fraction of the 700 PP inputs were randomly picked from the ${{P}_1}$ pattern and remaining $( {1 - \beta } )$ fraction of PP inputs were picked from pattern ${{P}_0}.$ We presented each of these 11 input patterns to the network and obtained the outputs of the granule cells in the network for each input pattern. We quantified pattern separation by plotting the similarity/dissimilarity among the granule cell outputs *vs*. the similarity/dissimilarity among the input patterns that were presented to elicit those outputs. This was repeated for each of the 11 patterns to cover a range of input similarities. Pattern separation was deemed to be implemented by the network if the similarity among the network outputs was lower than the similarity among the network inputs.^3,60^ All patterns were 2 s in duration. We allowed the network to stabilize for the first 500 ms and considered the remaining 1500 ms for computing similarity metrics.

### Similarity Metrics

We rigorously tested 6 different metrics, used in previous studies,^26,81,96,117^ to identify those that satisfied our 3 criteria: invariance to population size, average firing rate across the population, and the specific form of the firing rate distribution of input patterns. For all the metrics defined below, $N$ denoted the number of neurons in the population, ${{F}_{\beta ,i}}$ represented the normalized firing rate of ${{i}^{{\mathrm{th}}}}$ neuron of the ${{P}_\beta }\ $pattern. Hamming distance ($H{{D}_\beta }$) between ${{P}_0}\ $and ${{P}_\beta }$ was computed as


(12) \begin{eqnarray*}
H{{D}_\beta } = \mathop \sum \limits_{i = 1}^N \left| {{{F}_{0,i}} - {{F}_{\beta ,i}}} \right|.
\end{eqnarray*}

Normalized Euclidean Distance ($nE{{D}_\beta }$) between ${{P}_0}\ $and ${{P}_\beta }$ was computed as


(13) \begin{eqnarray*}
nE{{D}_\beta } = \sqrt {\frac{{\mathop \sum \nolimits_{i = 1}^N {{{\left( {{{F}_{0,i}} - {{F}_{\beta ,i}}} \right)}}^2}}}{N}}.
\end{eqnarray*}

Cosine similarity (${{\theta }_\beta }$) between ${{P}_0}\ $and ${{P}_\beta }$ was computed as


(14) \begin{eqnarray*}
{{\theta }_\beta } = \frac{{{{F}_0}\ \cdot \ {{F}_\beta }}}{{\left| {\left| {{{F}_0}\left| {\left| \ \right|} \right|{{F}_\beta }} \right|} \right|}},
\end{eqnarray*}

where ${{F}_\beta }$ represented the vector of average firing rate of ${{P}_\beta }$, ${{F}_0}\ \cdot \ {{F}_\beta }$ denoted the dot product between ${{F}_0}$ and ${{F}_\beta }$, and $| {| {{{F}_\beta }} |} |$ defined the ${{L}_2}$ norm of vector ${{F}_\beta }$.

Mutual information ($M{{I}_\beta }$) between ${{P}_0}\ $and ${{P}_\beta }$ was computed as


(15) \begin{eqnarray*}
M{{I}_\beta }\left( {{{F}_0},{{F}_\beta }} \right) &=& H\left( {{{F}_0}} \right) - H\left( {{{F}_0}{\mathrm{|}}{{F}_\beta }} \right)\\
&=& \mathop \sum \limits_{{{f}_0}\in{{F}_0}} \mathop \sum \limits_{{{f}_\beta }\in{{F}_\beta }} p\left( {{{f}_0},{{f}_\beta }} \right){{\log }_2}\frac{{p\left( {{{f}_0},{{f}_\beta }} \right)}}{{p\left( {{{f}_0}} \right)\ p\left( {{{f}_\beta }} \right)}},
\end{eqnarray*}

where ${{F}_\beta }$ defined the vector of average firing rate of ${{P}_\beta }$, $p({{f}_\beta })$ represented the marginal probability of firing rates in pattern ${{P}_\beta }$, and $p({{f}_0},{{f}_\beta })$ denoted the joint probability of firing rates in patterns ${{P}_0}$ and ${{P}_\beta }$.

The correlation metric between average firing rates (${{r}_\beta }$) between ${{P}_0}\ $and ${{P}_\beta }$ was defined as


(16) \begin{eqnarray*}
{{r}_\beta } = \textit{corr}\left( {{{F}_0},{{F}_\beta }} \right),
\end{eqnarray*}

where $corr()$ represented the Pearson correlation, and ${{F}_\beta }$ defined the vector built of the average firing rate of each neuron in pattern ${{P}_\beta }$.

To compute the correlation metric between instantaneous firing rates (${{R}_\beta }$) between ${{P}_0}\ $and ${{P}_\beta }$, we first computed the instantaneous firing rate of all the neurons when these two patterns were presented as network inputs. This was accomplished by convolving the spike train with a Gaussian function with $\mu = 0$ and $\sigma = \frac{1}{{10f}}$, where $f\ $was the average firing rate of the spike train. This resulted in $N$ rows corresponding to the instantaneous firing rates of $N$ neurons in each pattern. We then computed pairwise Pearson’s correlations between the instantaneous firing rates of all $N$ neurons for each of the two patterns under considerations, ${{P}_0}$ and ${{P}_\beta }.\ $We computed ${{R}_\beta }$ as the Pearson's correlation between the resulting $N \times N$ similarity matrices ${{C}_0}$ and ${{C}_\beta }$, associated respectively with ${{P}_0}$ and ${{P}_\beta }$.

To compare the dependence of these 6 similarity/dissimilarity metrics on the size of the population, we generated 3 independent sets of ${{P}_0}$ and ${{P}_1}$. The population size of the 3 sets of generated inputs were 1000, 2000, and 3000. We morphed each set of ${{P}_0}$–${{P}_1}$ pairs to generate 11 patterns for each set (3$ \times $11 patterns) and calculated all 6 metrics for 3 sets of morphed patterns. To assess the invariance of these metrics to average firing rates, we repeated a similar process involving 3 sets of ${{P}_0}$–${{P}_1}$ pairs with average firing rates of 5 Hz, 15 Hz, and 25 Hz. We considered Poisson vs. exponentially distributed firing frequencies to address the question of whether these metrics were invariant to the specific form of firing rate distribution. For each of the 3 factors (population size, average firing rate, and distribution type), we generated 10 independent sets of patterns and analyzed whether the mean similarity/dissimilarity varied across different conditions.

### Unbiased Stochastic Search in Synaptic Weights Space to Identify Heterogeneous Networks Performing Effective Pattern Separation

We generated one DG network capable of executing pattern separation by hand-tuning the 8 synaptic weights. However, as hand tuning yields a single biased network that does not account for network-to-network variability in synaptic connectivity, we resorted to searching for DG networks that were effective in performing pattern separation across the synaptic weight space. We modified the unbiased stochastic search (MPMOSS) strategy that we had used to identify valid single-neuron models to now search for valid network models that were effective in performing pattern separation. The parametric search space was defined by the 8 synaptic weights between the different cell types, which were picked randomly from respective uniform distributions for the underlying conductances (Supplementary Table S8). We generated 20 000 random networks by sampling the synaptic weights space and validated them against bounds on measurements that defined effective pattern separation (Figure 4C; Supplementary Table S9). As our search space was limited to the synaptic weight space, we used the same unique heterogeneous combinations of neuron subtypes used in the hand-tuned base model for all 20 000 randomly networks generated. In another set of simulations, we kept the synaptic weights to be fixed but randomly re-picked heterogeneous neurons from the respective populations, with neuronal choices different from our hand-tuned network (Supplementary Figure S6*B*). The population-of-networks approach involving unbiased random search spanning multiple parameters and multiple validation measurements allowed us to explore variability across different valid networks that performed pattern separation.

### Assessing the Impact of Individual Interneuron Subtypes on Pattern Separation in the Population of DG Networks

To assess the role of the specific interneuron subtypes (ie, basket cells, mossy cells, and HIPP cells) in pattern separation performance by the heterogeneous DG networks, we systematically deleted each interneuron population from the network and computed pattern separation metrics. For deletion of individual neuronal subtypes, we set the weights of all the synapses formed onto or by that interneuron subtype to zero. We repeated this procedure for each of the 3 interneuron subtypes for all ${{N}_{\textit{valid}}}$ number of valid DG networks (total $3 \times {{N}_{\textit{valid}}}$ networks). We plotted ${{S}_{out}}$ vs. ${{S}_{in}}$ spanning all 11 patterns for each of the $3 \times {{N}_{\textit{valid}}}$ DG networks, using both the ${{r}_\beta }$ and ${{R}_\beta }$ metrics. We computed the different pattern separation measurements for all these networks and compared these measurements with their base counterparts where all interneurons were intact.

### Assessing Robustness of the Pattern Separation Performance in the Heterogeneous and Homogeneous DG Network Population to Synaptic Jitter and Noise

We compared pattern separation performance of heterogeneous and homogeneous DG networks when perturbations were introduced into the synaptic weights and/or synaptic currents. We used the ${{N}_{\textit{valid}}}$ number of heterogeneous networks generated from the stochastic search to generate a population of homogeneous networks. We implemented this by replacing the non-repeating population of neurons in the heterogeneous networks by a homogeneous set of base neuron models. In other words, in the homogeneous network all GCs were identical to each other and so were each of the 3 different interneuron subtypes (one per subtype). This gave us 1 homogeneous network corresponding to each of the ${{N}_{\textit{valid}}}$ heterogeneous networks, with conserved synaptic connectivity between the homogeneous and heterogeneous counterparts.

We introduced perturbations to the network either as jitter to the synaptic weights of the network or as additive noise to the synaptic current or both. For synaptic weight perturbation, we introduced random jitter to each synaptic weight, picked from a Gaussian distribution with mean zero and standard deviation $\sigma $. We introduced 3 levels of jitter: low ($\sigma = 1$), medium ($\sigma = 3$), and high ($\sigma = 5$). We introduced additive zero-mean Gaussian noise to the synaptic current with 3 levels: low ($\sigma = 1$), medium ($\sigma = 3$), and high ($\sigma = 5$). When both jitter and noise were present together, we perturbed both the synaptic weight and added additive Gaussian noise, with the same 3 levels of perturbations. We computed the different pattern separation metrics using both the ${{r}_\beta }$ and ${{R}_\beta }$ metrics for all ${{N}_{\textit{valid}}}$ heterogeneous networks and their homogeneous counterparts to compare pattern separation performance between homogeneous vs. heterogeneous networks.

### Computational Details

All simulations and analyses were performed within the MATLAB programming environment (Mathworks Inc.) with custom written codes. The integration time step (${\mathrm{\Delta }}t$) was fixed at 0.1 ms for all the simulations. All statistical analyses were performed using R.^118^

## Supplementary Material

zqaf035_Supplementary

## Data Availability

The data underlying this article are available in the article and in its online supplementary material. The original source code used for the simulations reported in this article are publicly available at https://doi.org/10.5281/zenodo.16757590.
